# Genome-Wide QTL Mapping for Wheat Processing Quality Parameters in a Gaocheng 8901/Zhoumai 16 Recombinant Inbred Line Population

**DOI:** 10.3389/fpls.2016.01032

**Published:** 2016-07-19

**Authors:** Hui Jin, Weie Wen, Jindong Liu, Shengnan Zhai, Yan Zhang, Jun Yan, Zhiyong Liu, Xianchun Xia, Zhonghu He

**Affiliations:** ^1^National Wheat Improvement Center, Institute of Crop Science, Chinese Academy of Agricultural SciencesBeijing, China; ^2^Department of Plant Genetics & Breeding/State Key Laboratory for Agrobiotechnology, China Agricultural UniversityBeijing, China; ^3^Wheat and Maize Research Center, Institute of Cotton Research, Chinese Academy of Agricultural SciencesAnyang, China; ^4^International Maize and Wheat Improvement Center (CIMMYT) China officeBeijing, China

**Keywords:** high-density linkage map, Mixograph, Mixolab, QTL mapping, quality parameters, rapid visco-analyzer, single nucleotide polymorphism, *Triticum aestivum*

## Abstract

Dough rheological and starch pasting properties play an important role in determining processing quality in bread wheat (*Triticum aestivum* L.). In the present study, a recombinant inbred line (RIL) population derived from a Gaocheng 8901/Zhoumai 16 cross grown in three environments was used to identify quantitative trait loci (QTLs) for dough rheological and starch pasting properties evaluated by Mixograph, Rapid Visco-Analyzer (RVA), and Mixolab parameters using the wheat 90 and 660 K single nucleotide polymorphism (SNP) chip assays. A high-density linkage map constructed with 46,961 polymorphic SNP markers from the wheat 90 and 660 K SNP assays spanned a total length of 4121 cM, with an average chromosome length of 196.2 cM and marker density of 0.09 cM/marker; 6596 new SNP markers were anchored to the bread wheat linkage map, with 1046 and 5550 markers from the 90 and 660 K SNP assays, respectively. Composite interval mapping identified 119 additive QTLs on 20 chromosomes except 4D; among them, 15 accounted for more than 10% of the phenotypic variation across two or three environments. Twelve QTLs for Mixograph parameters, 17 for RVA parameters and 55 for Mixolab parameters were new. Eleven QTL clusters were identified. The closely linked SNP markers can be used in marker-assisted wheat breeding in combination with the Kompetitive Allele Specific PCR (KASP) technique for improvement of processing quality in bread wheat.

## Introduction

Protein and starch, the main components of wheat endosperm, play important roles in determining processing quality in bread wheat (*Triticum aestivum* L.). Gluten protein consists of glutenin and gliadin, responsible for dough rheological properties. Glutenin is divided into high- and low-molecular-weight glutenin subunits (HMW-GSs and LMW-GSs); these are encoded by *Glu-1* and *Glu-3* loci on chromosomes 1A, 1B, and 1D, respectively (Payne, 1987). Gliadins are encoded by *Gli-1* and *Gli-2* loci on wheat group 1 and 6 homoeologous chromosomes, respectively (Payne, 1987). It is generally agreed that subunits or alleles 1, 7+8, 5+10, *Glu-A3b, Glu-A3d, Glu-B3d, Glu-B3g, GliB1b, GliA2b*, and *GliB2c* conferred superior dough rheological properties (Metakovsky et al., 1997; He et al., 2005; Liu et al., 2005; Liang et al., 2010; Jin et al., 2013). Starch is comprised of amylose and amylopectin. The expression of granule-bound starch synthase (GBSS) has a significant effect on amylose content, which is encoded by *Wx-1* loci on chromosomes 7AS, 4AL, and 7DS (Nakamura et al., 1993). Several studies found that amylose content showed significantly negative correlation with RVA peak viscosity (PV) and breakdown (BD) while presented significantly positive correlation with pasting temperature (Varavinit et al., 2003; Hung et al., 2007; Blazek and Copeland, 2008).

A large variation in dough properties and starch pasting properties was revealed among wheat cultivars (Bhattacharya et al., 1997; Branlard et al., 2001; He et al., 2004, 2005). Although genotype, environment, and their interaction had significant influence on quality characteristics (Peterson et al., 1992; Ohm and Chung, 1999; Branlard et al., 2001; Bao et al., 2004; Zhang et al., 2005b), genetic factor contributed relatively larger phenotypic variation (Eagles et al., 2002; Miura et al., 2002; He et al., 2005; Patil et al., 2009). Therefore, it is feasible to improve wheat quality by genotype.

Dough rheological properties have a significant effect on flour end-use products, which was widely evaluated by Mixograph (Liu et al., 2005; Jin et al., 2013). Previous studies showed that the midline peak time (MPT), midline peak value (MPV), midline peak width (MPW), and midline 8 min band width (MTxW) were closely related to grain, bread-making and cookie-making qualities (Martinant et al., 1998; Ohm and Chung, 1999; Chung et al., 2001; Kaur et al., 2014). Starch pasting property measured by RVA is the most important trait because it is closely associated with Asian noodle quality (Zhang et al., 2005a). Mixolab developed by Chopin Technologies Company (Kahraman et al., 2008) performs a complex analysis of flour. It has capabilities of measuring properties of both flour protein and starch under conditions of mixing and heating, thus simulating the mechanical work that occurs during baking (Rosell et al., 2007). Several studies indicated that the dough rheological properties, starch pasting properties and end-use products such as bread, cake, and cookie could be evaluated by Mixolab (Kahraman et al., 2008; Ozturk et al., 2008; Koksel et al., 2009). More than 350 Mixolab units have been marketed and used in various countries in the last years.

Dough strength and starch pasting characteristics are quantitative traits controlled by multiple genes. Several QTLs for Mixograph (Huang et al., 2006; McCartney et al., 2006; Patil et al., 2009; Tsilo et al., 2011; Li et al., 2012; Deng et al., 2013; Zheng et al., 2013; Prashant et al., 2015) and RVA parameters (McCartney et al., 2006; Sun et al., 2008; Zhao et al., 2009; Deng et al., 2015) were reported using different populations. However, these reports were primarily based on simple sequence repeat (SSR) markers, resulting in relatively large genetic distances defining QTL and markers due to the limited numbers of such markers, which leads to difficulty for gene cloning based on QTL analysis and marker-assisted selection (MAS) in wheat breeding. Therefore, it is necessary to develop higher density linkage maps for dissecting the genetic factors associated with these complex traits. It is widely accepted that the major effect QTLs for Mixograph and RVA parameters are associated with HMW-GSs and *Wx* genes, respectively. However, the associations of many other genes involved in dough rheological and starch pasting properties, and the genetic basis of these complex traits have not been well-documented. In addition, no QTLs for Mixolab parameters have been reported so far.

Gaocheng 8901, a bread wheat variety developed by the Gaocheng Agricultural Research Institute in Hebei province, has excellent dough strength and exhibits outstanding bread-making quality. Zhoumai 16, developed by Zhoukou Academy of Agricultural Sciences in Henan province, is a high yielding, multiple disease resistant variety. Therefore, it was interesting to dissect the genetic components of yield and quality traits in these varieties. The aims of the present study were to: (1) construct a high-density linkage map with the 90 and 660 K SNP assays, (2) provide a comprehensive insight into genetic loci for dough rheological and starch pasting properties using a genome-wide QTL mapping approach, and (3) identify SNP markers closely linked to QTLs associated with quality traits for MAS in wheat breeding.

## Materials and methods

### Plant materials and field trials

One hundred and seventy-six F_2:6_ RILs for QTL analysis were developed from a cross between Gaocheng 8901 and Zhoumai 16 by single seed descent. Gaocheng 8901 was a strong gluten variety with alleles 1, 7+8, 5+10, *Glu-A3g, Glu-B3d, Wx-A1a, Wx-B1a, Wx-D1a, Pina-D1b, Pinb-D1a*, and *Pinb-2v2*, whereas Zhoumai 16 has weak gluten with alleles Null, 7+9, 2+12, *Glu-A3c*, 1B.1R, *Wx-A1a, Wx-B1a, Wx-D1a, Pina-D1a, Pinb-D1b*, and *Pinb-2v3*. The compositions of all alleles were confirmed in parents by molecular markers (Smith et al., 1994; Francis et al., 1995; Lafiandra et al., 1997; Nakamura et al., 2002; Ma et al., 2003; Chen et al., 2006, 2010a,b; Lei et al., 2006; Liu et al., 2008; Saito et al., 2009; Wang et al., 2009, 2010). The parents and 176 RILs were planted at Anyang in Henan province during the 2012–2013 and 2013–2014 cropping seasons and at Suixi in Anhui province during 2013–2014 in triple replicated, randomized complete blocks. Each plot comprised four 1.5 m rows spaced 20 cm apart and about 50 seeds were sown in each row. Field management was according to local practice, and no sprouting of harvested seed was evident.

### Milling

Harvested grain was cleaned, and all samples had falling numbers >300 s. Kernel hardness was measured on 300-kernel samples using a Single Kernel Characterization System (SKCS 4100, Perten, Sweden). Kernel moisture and protein were determined with a near infrared transmittance (NIT) analyzer (Foss-Tecator 1241, Foss, Högänas, Sweden). Samples were tempered overnight to 14, 15, and 16% moisture contents for soft (SKCS hardness index (HI) < 40), medium (HI, 40-59), and hard (HI > 60) types, respectively. All samples were milled using a Brabender Quadrumat Junior Mill (Brabender Inc., Duisberg, Germany). Flour extraction rates were about 60%.

### Evaluation of flour properties with Mixograph, rapid visco-analyzer, and Mixolab

A 10-g Mixograph (National Mfg. Co., Lincoln, NE) was used to assess dough mixing characteristics and MPT, MPV, MPW, and MTxW were measured according to AACC (2000) method 54-40A. A RVA (Newport Scientific, Australia) was employed to evaluate the starch pasting properties of flour samples and PV, trough viscosity (TV), BD, final viscosity (FV), setback (SB), and peak time (PTI) were scored following AACC (2000) method 76-21 with minor modifications, viz., the reaction solution of water was replaced by 170 mg/L AgNO_3_ to eliminate the effect of α-amylase activity in flour on starch pasting properties.

A Mixolab (Chopin Technologies, France) was used to determine dough mixing and pasting properties of wheat flour simultaneously during dough mixing. About 50-g of flour was put into the Mixolab bowl and an appropriate amount of water was added to ensure that the torque of the dough was in the 1.1 ± 0.07 Nm range. Processing was divided into five stages based on the “Chopin 12heat” protocol as follows: establishing equilibrium at 30°C for 8 min, then heating to 90°C at a rate of 4°C/min for 15 min, holding at 90°C for 12 min, cooling to 50°C at a rate of 4°C/min for 10 min, and finally holding at 50°C for 5 min. The mixing speed was kept constant at 80 rpm. The parameters water absorption (WA), development time (DT), stability time (ST), C1 (the torque of maximum point in the first mixing stage), C2-C5 (the torque of end points in the corresponding mixing stages) were recorded during the procedure.

### SNP genotyping and functional marker analyses

A 90 K iSelect SNP array containing 81,587 markers and a 660 K SNP array with 630,517 markers were used to genotype all 176 RILs and 59 randomly selected RILs, respectively, at CapitalBio Corporation (Beijing, China; http://www.capitalbio.com). Genotypic clusters involving each SNP were confirmed using the polyploidy version of GenomeStudio software (Illumina, http://www.illumina.com).

Twenty-one functional markers identifying glutenin subunits, the 1B.1R translocation, kernel hardness and waxy alleles were used to screen the parents, and polymorphic markers were subsequently used to test the RILs. Sequences of PCR primers and fragment sizes are listed in Table S1.

### Construction of a high-density linkage map, QTL analysis, and identification of candidate genes

SNPs of poor quality, or with more than 10% of missing data, or segregation distortion of more than 0.35 were removed. Three procedures were followed in constructing the high-density linkage map. Firstly, among 81,587 SNPs (90 K) used in screening all 176 RILs, 12,205 were polymorphic between the parents. Of those, 2080 had more than 10% missing data, and 828 were not anchored to the linkage map. Finally, 9297 SNPs were used to construct a linkage map (defined as Map 1) using Joinmap 4.0 software (Stam, 1993; http://www.kyazma.nl). However, many SNPs were mapped at the same loci or within 0.01 cM among them. Therefore, BIN-Mapping function from IciMapping 4.0 (Li et al., 2007; http://www.isbreeding.net) was used to construct a skeleton map containing 2375 markers for subsequent QTL analysis. Secondly, among 90 and 660 K SNP analyses of 59 random RILs, 12,205 and 109,209, respectively, were polymorphic between the parents. Of those, 4809 and 20,498, respectively, had more than 10% missing data, and 2081 and 31,371, respectively, were not anchored to the linkage map. Thus, 5315 and 57,340 from two arrays, respectively, were used to construct the linkage map (defined as Map 2) with Joinmap 4.0 software; this was used to enrich the markers in several QTL regions with larger marker intervals from Map 1. Thirdly, Maps 1 and 2 were integrated into a high-density linkage map with MergeMap Online (Wu et al., 2011; http://www.mergemap.org).

The short and long chromosome arms of each linkage group were confirmed according to the wheat 90 and 660 K consensus SNP maps. The linkage map was constructed using MapChart 2.2 (Voorrips, 2002; http://www.earthatlas.mapchart.com). QTL analysis was carried out with QTL Cartographer 2.5 using composite interval mapping (Wang et al., 2007; http://statgen.ncsu.edu/qtlcart/WQTLCart.htm) based on Map 1. Logarithm of odds (LOD) scores ranged from 1.8 to 2.6 for all traits tested according to 2000 permutations tests at a probability of 0.01. Therefore, a LOD score of 2.6 was used for declaring significant QTL. The QTL × Environment (QE) interaction was analyzed by IciMapping 4.0 using the multi-environment trials (MET) (Li et al., 2007; http://www.isbreeding.net), with a LOD threshold based on 1000 permutation tests at a probability of 0.01. For the 90 K iSelect SNP genotyping assay, candidate genes were confirmed following Zhai et al. (2016). For the 660 K iSelect SNP genotyping assay, the sequences of flanking SNP markers tightly linked to QTL were blasted in the wheat genomes database (http://plants.ensembl.org/Triticum_aestivum/Info/Index) to determine SNP markers corresponding to the original genes, and these gene sequences were used as queries to blast the NCBI database (http://www.ncbi.nlm.nih.gov/) to identify putative gene functions. BLAST hits were filtered to an *e*-value threshold of 10^−5^ with an identity higher than 75%. Collinearity analysis was conducted by Ensembl Plants database (http://plants.ensembl.org/index.html) and blast hits were filtered with an *e*-value threshold of 10^−16^.

### Statistical analysis

Analyses of variance (ANOVA) and correlation were performed by SAS 9.0 (SAS Institute Inc., Cary, NC, USA). ANOVA was conducted using the PROC MIXED procedure, where environments were treated as fixed effects, and lines, line × environment interactions and replicates nested in environments were all treated as random effects. The broad-sense heritabilities ($h_{B}^{2}$) of all the traits were calculated by $h_{B}^{2}=\sigma_{g}^{2}/\left(\sigma_{g}^{2}+\sigma_{ge}^{2}/r+\sigma_{\varepsilon }^{2}/re\right)$, where $\sigma_{g}^{2},\;\sigma_{ge}^{2}$ and $\sigma_{\varepsilon }^{2}$ were estimates of line, line × environment interaction and residual error variances, respectively, and *e* and *r* represented the numbers of environments and replicates, respectively. Pearson's correlation coefficients were calculated between traits with the PROC CORR procedure based on mean values across three environments.

## Results

### Phenotypic description

Gaocheng 8901 performed much better than Zhoumai 16 in Mixograph parameters and Mixolab dough rheological scores based on data in three environments (Table 1), whereas Zhoumai 16 showed better performance than Gaocheng 8901 for RVA parameters and Mixolab starch pasting properties. Wide variations were observed for all traits in the RIL population (Table 1). The frequency distribution for all traits indicated continuous variation, with transgressive segregation (Figure S1), indicating polygenic inheritance.

**Table 1:** **Means, standard deviations, and ranges of quality traits of the parents and RILs in three environments**.

**Traits**	**Environments**	**Parents**		**RILs**	
		**Gaocheng 8901**	**Zhoumai 16**	**Mean ± SD**	**Range**
MPT (min)	Anyang 2012–2013	5.1	2.3	3.2 ± 0.8	1.7 ~ 6.2
	Anyang 2013–2014	4.1	2.6	3.1 ± 0.7	1.8 ~ 5.0
	Suixi 2013–2014	4.1	2.2	3.1 ± 0.7	1.7 ~ 4.9
	Average	4.4	2.4	3.1 ± 0.7	1.9 ~ 4.8
MPV (%)	Anyang 2012–2013	54.3	48.2	51.9 ± 3.9	43.1 ~ 61.0
	Anyang 2013–2014	52.2	46.6	49.7 ± 3.4	40.1 ~ 59.9
	Suixi 2013–2014	52.8	45.9	51.3 ± 3.5	42.9 ~ 62.0
	Average	53.1	46.2	51.0 ± 3.3	43.3 ~ 60.2
MPW (%)	Anyang 2012–2013	22.9	21.6	22.1 ± 3.2	14.3 ~ 34.8
	Anyang 2013–2014	17.9	14.3	17.4 ± 2.6	11.1 ~ 25.1
	Suixi 2013–2014	21.6	14.3	18.8 ± 3.2	12.7 ~ 34.9
	Average	20.8	16.7	19.5 ± 2.5	13.7 ~ 31.2
MTxW (%)	Anyang 2012–2013	18.0	3.4	3.7 ± 6.5	2.6 ~ 19.7
	Anyang 2013–2014	5.5	3.3	4.6 ± 1.4	2.8 ~ 11.7
	Suixi 2013–2014	10.0	3.1	4.9 ± 2.1	2.8 ~ 14.2
	Average	11.1	3.3	5.3 ± 2.2	2.8 ~ 13.1
PV (RVU)	Anyang 2012–2013	277.8	302.9	279.7 ± 19.8	204.8 ~ 321.7
	Anyang 2013–2014	255.0	280.3	260.8 ± 18.5	197.0 ~ 326.9
	Suixi 2013–2014	223.1	270.6	243.5 ± 26.4	146.6 ~ 326.5
	Average	252.1	284.6	261.2 ± 18.2	202.4 ~ 318.1
TV (RVU)	Anyang 2012–2013	199.8	207.7	195.9 ± 17.2	133.8 ~ 245.5
	Anyang 2013–2014	203.9	240.1	200.1 ± 13.8	140.0 ~ 236.3
	Suixi 2013–2014	175.5	208.3	185.3 ± 21.4	97.5 ~ 253.8
	Average	193.1	203.4	193.8 ± 13.3	142.3 ~ 244.7
BD (RVU)	Anyang 2012–2013	78.0	95.3	83.8 ± 13.4	54.8 ~ 121.0
	Anyang 2013–2014	51.2	86.2	60.7 ± 11.6	37.3 ~ 101.3
	Suixi 2013–2014	47.6	62.3	58.2 ± 11.1	32.9 ~ 100.0
	Average	59.0	81.2	67.4 ± 10.2	44.1 ~ 102.2
FV (RVU)	Anyang 2012–2013	332.0	362.7	333.9 ± 20.3	263.7 ~ 381.3
	Anyang 2013–2014	318.0	346.4	323.6 ± 16.1	277.0 ~ 359.4
	Suixi 2013–2014	272.9	339.8	299.0 ± 30.9	161.9 ~ 375.4
	Average	307.7	349.7	318.8 ± 17.8	264.9 ~ 363.5
SB (RVU)	Anyang 2012–2013	132.2	150.0	138.1 ± 15.3	106.0 ~ 180.5
	Anyang 2013–2014	114.1	152.2	123.5 ± 10.6	96.1 ~ 159.4
	Suixi 2013–2014	97.4	131.5	113.8 ± 14.1	64.4 ~ 155.9
	Average	114.6	146.3	125.0 ± 11.2	100.5 ~ 161.9
PTI (min)	Anyang 2012–2013	6.97	7.00	6.95 ± 0.07	6.67 ~ 7.00
	Anyang 2013–2014	6.83	7.00	6.84 ± 0.11	6.43 ~ 7.00
	Suixi 2013–2014	6.60	6.90	6.76 ± 0.14	6.33 ~ 7.00
	Average	6.80	6.97	6.85 ± 0.09	6.53 ~ 7.00
WA (%)	Anyang 2012–2013	66.0	60.8	64.4 ± 2.7	56.7 ~ 70.7
	Anyang 2013–2014	65.2	61.0	64.0 ± 2.1	57.0 ~ 68.4
	Suixi 2013–2014	65.0	61.6	64.0 ± 2.2	57.0 ~ 68.5
	Average	65.4	61.1	64.2 ± 2.2	56.9 ~ 69.0
DT (min)	Anyang 2012–2013	7.8	2.5	3.8 ± 1.3	1.5 ~ 8.1
	Anyang 2013–2014	4.6	2.1	3.5 ± 1.0	1.8 ~ 7.7
	Suixi 2013–2014	5.4	2.1	3.5 ± 1.0	1.9 ~ 7.6
	Average	5.9	2.2	3.6 ± 1.0	1.7 ~ 7.7
ST (min)	Anyang 2012–2013	11.1	4.3	7.3 ± 2.1	2.4 ~ 11.6
	Anyang 2013–2014	7.1	3.2	5.1 ± 1.8	1.7 ~ 10.1
	Suixi 2013–2014	9.2	3.1	5.8 ± 1.8	2.3 ~ 10.7
	Average	9.2	3.5	6.1 ± 1.8	2.1 ~ 10.5
C2 (Nm)	Anyang 2012–2013	0.49	0.39	0.41 ± 0.07	0.19 ~ 0.58
	Anyang 2013–2014	0.50	0.41	0.43 ± 0.06	0.31 ~ 0.60
	Suixi 2013–2014	0.47	0.37	0.40 ± 0.06	0.23 ~ 0.58
	Average	0.49	0.39	0.42 ± 0.06	0.27 ~ 0.56
C3 (Nm)	Anyang 2012–2013	1.81	–	1.67 ± 0.20	1.09 ~ 2.31
	Anyang 2013–2014	1.91	1.96	1.86 ± 0.17	1.48 ~ 2.50
	Suixi 2013–2014	1.85	1.77	1.70 ± 0.18	1.30 ~ 2.49
	Average	1.85	1.86	1.75 ± 0.18	1.34 ~ 2.43
C4 (Nm)	Anyang 2012–2013	1.55	–	1.48 ± 0.28	0.68 ~ 2.25
	Anyang 2013–2014	1.79	1.84	1.74 ± 0.17	1.31 ~ 2.39
	Suixi 2013–2014	1.56	1.71	1.50 ± 0.25	0.78 ~ 2.42
	Average	1.63	1.78	1.58 ± 0.20	1.04 ~ 2.35
C5 (Nm)	Anyang 2012–2013	2.18	2.88	2.19 ± 0.48	0.99 ~ 3.77
	Anyang 2013–2014	2.75	3.03	2.78 ± 0.36	1.98 ~ 3.85
	Suixi 2013–2014	2.20	2.61	2.21 ± 0.48	1.01 ~ 3.86
	Average	2.38	2.84	2.39 ± 0.38	1.57 ~ 3.83

*Average, Pooled data for three environments; BD, RVA breakdown; C2, Mixolab protein weakening torque; C3, Mixolab starch gelatinization peak torque; C4, Mixolab starch gelatinization trough torque; C5, Mixolab starch gelatinization final torque; DT, Mixolab development time; FV, RVA final viscosity; MPT, Mixograph midline peak time; MPV, Mixograph midline peak value; MPW, Mixograph midline peak width; MTxW, Mixograph midline 8 min band width; PTI, RVA peak time; PV, RVA peak viscosity; SB, RVA setback; SD, Standard deviation; ST, Mixolab stability time; TV, RVA trough viscosity; WA, Mixolab water absorption; “–” indicated no available data*.

ANOVA showed that genotype, environment and G × E interaction had significant effects on all traits except for MPW and PTI. Genotype and environment showed significant effects on MPW and PTI. Broad-sense heritabilities based on plot means ranged from 0.56 to 0.94 (Table 2).

**Table 2:** **Analysis of variance and broad-sense heritabilities for quality traits in RILs based on three environments**.

**Traits**	**Mean squares**				**$h_{B}^{2}$**
	**Genotype**	**Environment**	**G × E**	**Error**	
MPT	1.4^***^	0.5^***^	0.1^***^	0.1	0.92
MPV	41.3^***^	245.0^***^	4.1^***^	2.1	0.90
MPW	27.5^***^	916.3^***^	6.1	5.1	0.78
MTxW	15.5^***^	46.2^***^	2.3^***^	1.1	0.91
PV	140502^***^	5348194^***^	40821^***^	22002	0.71
TV	78552^***^	744184^***^	34227^*^	25282	0.56
BD	55818^***^	3628868^***^	15993^**^	10794	0.71
FV	134286^***^	5256910^***^	58740^**^	41070	0.56
SB	63694^***^	2835448^***^	19696^**^	12980	0.69
PTI	0.04^***^	1.26^***^	0.01	0.01	0.72
WA	18.2^***^	12.7^***^	1.4^***^	0.6	0.92
DT	2.9^***^	2.9^***^	0.5^***^	0.3	0.83
ST	9.7^***^	155.2^***^	1.3^***^	0.5	0.87
C2	0.010^***^	0.049^***^	0.002^***^	0.001	0.76
C3	0.10^***^	1.59^***^	0.01^***^	0.01	0.86
C4	0.14^***^	3.34^***^	0.05^***^	0.02	0.94
C5	0.5^***^	20.5^***^	0.1^***^	0.0	0.71

* *Significant at P < 0.05*.

** *Significant at P < 0.01*.

*** *Significant at P < 0.001*.

*See footnote of Table 1 for abbreviations*.

The significant and positive correlations were found among MPT, DT, ST, and C2 (Table 3). MPV and WA were significantly and positively correlated (*r* = 0.62). MTxW was significantly correlated with Mixolab parameters ST, C2, C3, C4, and C5.

**Table 3:** **Correlation coefficients among Mixograph, RVA, and Mixolab parameters averaged across environments**.

	**MPT**	**MPV**	**MPW**	**MTxW**	**PV**	**TV**	**BD**	**FV**	**SB**	**PTI**	**WA**	**DT**	**ST**	**C2**	**C3**	**C4**	**C5**
MPT	1																
MPV	ns	1															
MPW	ns	0.82^***^	1														
MTxW	0.71^***^	ns	ns	1													
PV	ns	ns	ns	ns	1												
TV	ns	ns	ns	ns	0.84^***^	1											
BD	ns	ns	ns	ns	0.70^***^	ns	1										
FV	ns	ns	ns	ns	0.90^***^	0.78^***^	0.61^***^	1									
SB	ns	ns	ns	ns	0.45^***^	ns	0.67^***^	0.67^***^	1								
PTI	ns	ns	ns	ns	0.32^***^	ns	0.68^***^	0.37^***^	0.69^***^	1							
WA	ns	0.62^***^	0.34^***^	ns	ns	ns	ns	ns	ns	ns	1						
DT	0.80^***^	ns	0.36^***^	ns	ns	ns	ns	ns	ns	ns	ns	1					
ST	0.85^***^	ns	ns	0.83^***^	ns	ns	ns	ns	ns	ns	ns	0.82^***^	1				
C2	0.60^***^	ns	ns	0.75^***^	ns	ns	ns	ns	ns	ns	ns	0.62^***^	0.80^***^	1			
C3	ns	ns	ns	0.54^***^	ns	ns	ns	0.31^***^	ns	ns	–0.70^***^	ns	0.53^***^	0.65^***^	1		
C4	ns	ns	ns	0.43^***^	ns	ns	ns	0.44^***^	0.35^***^	ns	–0.60^***^	ns	0.43^***^	0.62^***^	0.92^***^	1	
C5	ns	ns	ns	0.37^***^	ns	ns	ns	0.38^***^	0.39^***^	ns	–0.65^***^	ns	0.36^***^	0.61^***^	0.89^***^	0.93^***^	1

*** *Significant at P < 0.001, ns, not significant at P < 0.05*.

*See footnote of Table 1 for abbreviations*.

### High-density linkage map

The high-density linkage map comprised 8067 (90 K) and 38,894 (660 K) SNP markers, and spanned a total length of 4121 cM involving all 21 chromosomes, with an average chromosome length of 196.2 cM, ranging from 78.0 cM (3D) to 387.5 cM (5B) (Tables S2, S3). The A genome included 20,012 SNPs (42.6%) covering a length of 1439.8 cM and an average marker density of 0.07 cM; the B genome had 22,142 SNPs (47.1%) covering 1783.6 cM and an average marker density of 0.08 cM; the D genome included 4807 SNPs (10.2%), a length of 897.7 cM, and an average marker density of 0.19 cM. The number of SNP markers in each chromosome ranged from 43 (5D) to 6042 (4A). The marker intervals ranged from 0.02 (4A) to 2.14 (5D) cM, with a means of 0.09 cM.

### QTLs for quality traits

A total of 119 additive QTLs were identified for 17 quality parameters using the 90 K iSelect SNP genotyping assay linkage map; 15 stable QTLs explained more than 10% of the phenotypic variation across environments (Table 4). In addition, 39 QTLs exhibited significant interaction with environments (Table 4).

**Table 4:** **QTLs for quality traits and QTL × environment interaction detected in the Gaocheng 8901/Zhoumai 16 population based on the linkage map from the 90 K iSelect SNP genotyping assay**.

**Single environment analysis**							**Multiple-environment analysis**			
**QTL**	**Environment**	**Closest marker**	**Distance (cM)**	**LOD**	**Add**	***R^2^* (%)**	**Add**	**AbyE_I**	**AbyE_II**	**AbyE_III**
**MIXOGRAPH MIDLINE PEAK TIME (MPT)**										
*QMPT.caas.1AS*	I, II	*BS00074517_51*	0.03	2.6~3.5	−0.23~–0.25	5.9~7.5				
*QMPT.caas.1BL*	I, II, III, A	*wsnp_Ra_c7527_12935330*	0.05	8.7~14.5	−0.26~–0.36	6.6~11.3	−0.07	0.05	−0.12	0.07
*QMPT.caas.1DL*	I, II, III, A	*GENE*–*0511_484*	0.02	22.7~29.6	−0.44~–0.53	37.6~52.6	−0.48	−0.07	0.05	0.01
*QMPT.caas.2BS.1*	I, II, III, A	*BS00109268_51*	0.03	2.8~3.4	−0.18~–0.21	5.8~6.9				
*QMPT.caas.2BS.2*	I, II, A	*Tdurum_contig65756_350*	0.04	3.1~4.5	−0.25~–0.30	6.5~9.9				
*QMPT.caas.4BS*	II, III	*BS00022534_51*	0.06	3.6~4.4	−0.18~–0.21	6.7~8.1	−0.14	−0.05	0.05	0.00
*QMPT.caas.4BL*	II, III, A	*RAC875_rep_c72961_977*	0.02	4.6~6.4	0.21~0.29	9.5~13.6				
QMPT.caas.5AL^*^	I, II, III, A	*wsnp_CAP11_c1740_947838*	1.00	2.6~3.0	0.21~0.25	6.9~8.4				
*QMPT.caas.7AS*	I, III	*wsnp_CAP12_c3056_1439567*	0.07	2.7~3.7	−0.17~–0.23	6.2~8.2				
QMPT.caas.7DL^*^	I, II, III, A	*Excalibur_c30913_512*	0.02	2.8~3.3	0.17~0.20	6.3~7.8				
**MIXOGRAPH MIDLINE PEAK VALUE (MPV)**										
*QMPV.caas.1AL*	I, II, III, A	*Glu*–*A1*	1.03	7.8~11.3	−1.50~–2.07	15.8~21.9	−0.60	0.51	−0.92	0.41
*QMPV.caas.1BL*	I, II, III, A	*H20*	0.05	19.7~30.3	−1.80~–1.95	13.4~21.6	−1.61	0.00	0.03	−0.04
*QMPV.caas.1DL*	I, III, A	*RAC875_c44639_317*	0.05	3.1~3.8	1.00~1.01	7.5~8.5	0.54	0.54	−0.20	−0.34
QMPV.caas.3B^*^	I, II, A	*BS00106922_51*	0.01	5.8~6.9	−1.70~–2.10	13.2~14.9				
QMPV.caas.3BL^*^	II, III, A	*TA003797*–*0888*	0.03	4.5~5.2	1.15~1.18	10.8~11.3				
QMPV.caas.5AL^*^	I, II, III, A	*Tdurum_contig44343_1039*	1.06	3.7~4.9	−1.26~–1.57	9.8~12.8				
*QMPV.caas.5BL*	II, III, A	*IAAV5469*	0.02	2.6~4.0	0.89~1.36	5.9~9.5				
*QMPV.caas.6AS*	I, II, A	*BS00022628_51*	0.00	3.1~3.7	−0.92~–1.05	6.7~7.6				
*QMPV.caas.6AL*	I, III, A	*BobWhite_c3714_659*	0.02	3.2~3.9	−1.44~–1.46	7.0~9.9				
QMPV.caas.6BL^*^	I, II, III, A	*Kukri_c61680_247*	0.07	2.9~4.4	1.09~1.40	6.5~10.0				
*QMPV.caas.7AS*	I, II, III	*RAC875_c4889_1393*	0.00	2.6~4.1	0.91~1.14	6.3~9.1	0.54	−0.19	0.06	0.13
**MIXOGRAPH MIDLINE PEAK WIDTH (MPW)**										
*QMPW.caas.1AL*	I, II, III, A	*Glu*–*A1*	1.03	8.0~10.4	−1.47~–1.48	18.3~21.6	−0.95	−0.46	0.97	−0.51
*QMPW.caas.1BL*	I, II, III, A	*H20*	0.05	8.0~21.4	−1.01~–1.51	6.1~15.8	−0.77	0.03	0.49	−0.51
QMPW.caas.2BL.1^*^	I, II, A	*Excalibur_c74466_344*	0.02	4.4~4.5	−1.10~–1.30	10.1~10.4				
QMPW.caas.2BL.2^*^	I, II	*IACX45*	0.40	2.9~3.8	0.98~1.30	8.5~9.5				
*QMPW.caas.3B*	II, III, A	*Kukri_rep_c73303_688*	0.03	2.7~4.0	−0.79~–1.02	5.8~8.4				
*QMPW.caas.5AL*	II, III	*RAC875_c23340_2243*	0.80	2.6~2.9	0.77~1.04	6.7~6.8				
QMPW.caas.5BS^*^	I, II	*CAP11_c2608_130*	0.90	3.1	0.86~0.92	6.7~7.2				
QMPW.caas.7AL^*^	II, III, A	*Tdurum_contig57301_395*	0.06	2.6~5.0	0.83~1.00	5.9~11.1	−0.32	0.33	−0.56	0.23
**MIXOGRAPH MIDLINE TIME 8 WIDTH (MTxW)**										
*QMTXW.caas.1BL*	I, II, A	*Tdurum_contig43910_1113*	0.01	8.6~17.4	−0.58~–1.12	6.0~12.4	−0.74	−0.53	0.14	0.39
*QMTXW.caas.1DL*	I, II, III, A	*GENE-0511_484*	0.02	3.4~5.1	−0.42~–1.37	8.2~12.0	−0.66	−0.51	0.27	0.24
*QMTXW.caas.2AS*	I, III, A	*wsnp_CAP8_c1580_908907*	0.02	2.8~3.2	−0.39~–0.62	7.2~8.5	−0.42	−0.16	0.19	−0.02
QMTxW.caas.2BS^*^	I, II, III, A	*Excalibur_c84424_334*	0.72	2.6~4.4	−0.52~–1.12	5.9~10.1				
*QMTxW.caas.4B*	I, II, III, A	*RAC875_c65971_127*	0.01	3.0~4.9	−0.40~–1.49	6.9~11.0	−0.54	−0.62	0.25	0.36
QMTxW.caas.5BL^*^	II, III, A	*TA002756-0960*	0.03	2.6~4.1	0.51~0.76	5.3~9.8				
**RVA PEAK VISCOSITY (PV)**										
*QPV.caas.5BL*	I, III, A	*Excalibur_c28091_279*	0.50	2.6~3.5	−62.25~–101.11	5.3~8.8				
*QPV.caas.6AL*	II, III	*wsnp_Ex_c34597_42879693*	0.46	2.7~2.8	58.55~86.21	6.5~6.9				
*QPV.caas.6BL.1*	I, II	*D_contig19487_399*	0.00	2.6~3.1	−77.55~–78.17	6.3~7.2				
*QPV.caas.6BL.2*	I, II, A	*RAC875_c50348_93*	5.00	3.3~5.7	86.47~103.09	10.3~12.7				
*QPV.caas.7BL*	II, III, A	*RAC875_c62671_1019*	1.00	2.8~3.6	−57.80~–85.70	6.4~10.0	−48.66	1.66	−4.81	3.14
**RVA TROUGH VISCOSITY (TV)**										
QTV.caas.1BL^*^	I, II, A	*Kukri_c7770_176*	0.02	9.9~23.8	−60.60~–87.10	7.6~17.9				
QTV.caas.5BL.1^*^	I, II, A	*Kukri_c6717_2903*	0.01	3.5~4.7	45.09~63.35	7.6~9.9				
QTV.caas.5BL.2^*^	I, II, A	*Excalibur_c51744_536*	1.98	2.6~4.0	−47.54~–67.10	5.7~10.3				
QTV.caas.5BL.3^*^	I, III, A	*BS00039874_51*	0.04	2.7~3.9	62.01~73.78	6.1~8.1				
QTV.caas.6BS^*^	I, II	*Kukri_c48571_361*	0.03	2.7~4.5	−52.32~–73.60	6.7~10.5				
**RVA BREAKDOWN (BD)**										
QBD.caas.5AS^*^	I, III, A	*Excalibur_c51706_263*	0.04	3.2~4.5	37.83~45.77	6.8~10.6	30.30	−8.38	9.24	−0.85
QBD.caas.7DL^*^	I, II, III, A	*Excalibur_c4508_1007*	0.01	3.0~6.7	−41.70~–53.38	10.4~14.0	−38.44	−0.40	−1.67	2.07
**RVA FINAL VISCOSITY (FV)**										
QFV.caas.5AS^*^	I, II, A	*RFL_Contig2251_434*	0.05	3.3~4.4	67.37~92.30	7.6~10.5				
QFV.caas.5AL^*^	I, II	*Excalibur_c7180_862*	0.17	3.1~3.5	−59.57~–85.19	7.2~8.0				
*QFV.caas.5BL.1*	I, II	*Kukri_c6717_2903*	0.01	2.6~4.8	49.95~73.32	6.2~9.5				
*QFV.caas.5BL.2*	I, III, A	*BS00031339_51*	0.01	4.3~6.7	−103.30~–143.61	9.9~13.6				
QFV.caas.7DL^*^	I, II	*Excalibur_c4508_1007*	0.01	2.7~3.5	−57.24~–71.05	6.5~8.6				
**RVA SETBACK (SB)**										
QSB.caas.1AL^*^	II, III	*wsnp_Ex_c1427_2736441*	0.02	2.6~2.9	−35.06~–43.44	6.2~6.4				
QSB.caas.5BL^*^	I, III	*Jagger_c4951_122*	0.04	2.6~5.1	−43.16~–62.77	5.8~11.1				
*QSB.caas.6BL*	II, III, A	*Excalibur_c64024_119*	0.90	2.8~3.4	−40.82~–48.73	6.8~8.8				
**RVA PEAK TIME (PTI)**										
QPTI.caas.1BL^*^	I, III, A	*BS00022124_51*	0.02	9.8~10.9	0.03~0.04	6.7~8.5	0.03	−0.02	0.00	0.02
QPTI.caas.2AL^*^	I, II, III, A	*Excalibur_c6710_2192*	0.00	2.8~5.7	0.02~0.04	6.4~12.5				
QPTI.caas.3B^*^	I, III, A	*BS00023645_51*	0.02	2.7~4.5	0.02~0.05	6.1~10.3				
QPTI.caas.3BL^*^	II, III, A	*wsnp_Ex_c36937_44788679*	0.07	3.7~5.7	−0.03~–0.05	7.9~11.6	−0.02	0.01	0.02	−0.03
*QPTI.caas.3DL*	I, II, A	*Kukri_c24037_162*	1.05	4.7~6.9	−0.04~–0.05	13.0~16.6				
*QPTI.caas.4AS*	I, II, III, A	*RAC875_rep_c106151_123*	0.03	3.6~5.8	0.03–0.05	8.3~12.1				
*QPTI.caas.4AL*	I, III, A	*Excalibur_c20818_585*	0.01	3.5~4.2	0.02–0.05	7.7~8.9				
QPTI.caas.5BL^*^	I, II	*BS00071207_51*	0.02	2.7~3.8	−0.02~–0.05	6.4~8.9				
*QPTI.caas.7DS*	II, III	*wsnp_BE490643D_Ta_2_1*	0.04	2.6~5.1	−0.04	6.6~12.1	−0.02	0.01	−0.01	0.00
**MIXOLAB WATER ABSORPTION (WA)**										
*QWA.caas.1AL*	I, II, III, A	*IACX5793*	0.02	4.4~8.0	−0.89~–0.98	10.3~17.7	−0.66	0.28	−0.15	−0.13
*QWA.caas.2AS*	I, II	*Excalibur_rep_c105284_131*	0.08	3.0~3.6	0.67~1.03	6.3~8.0				
*QWA.caas.2BS*	II, III	*Kukri_c60090_82*	0.06	2.6~3.0	−0.65~–0.67	6.0~7.2				
*QWA.caas.4AL*	I, III	*Excalibur_c15280_1242*	0.18	2.6	−0.56~–0.70	5.4~5.5				
*QWA.caas.4BS*	II, III	*RAC875_c16017_1107*	0.01	2.7	0.53~0.57	5.9~6.2				
*QWA.caas.5DS*	I, II, III, A	*Ha*	0.00	3.6~5.3	−0.66~–1.00	8.0~11.8	−0.75	−0.40	0.15	0.25
*QWA.caas.6A*	II, III, A	*BS00067934_51*	0.05	2.6~3.7	−0.56~–0.62	7.5~7.9				
*QWA.caas.6BS*	I, II, III, A	*Tdurum_contig64467_233*	0.07	3.1~4.9	0.70~0.78	6.4~10.6	0.53	−0.19	0.05	0.14
*QWA.caas.6BL*	I, III	*GENE-1074_108*	0.03	2.7~3.7	0.70~0.81	6.3~8.3				
*QWA.caas.7AS*	I, II, III, A	*Excalibur_c9183_1397*	0.00	3.9~5.0	−0.82~–1.10	8.6~11.5				
*QWA.caas.7AL*	I, II, III, A	*wsnp_Ex_rep_c67593_66232317*	0.03	2.9~4.5	0.71~0.83	6.3~9.5				
*QWA.caas.7BL*	I, II, III, A	*Tdurum_contig59755_568*	0.04	3.5~4.6	−0.83~–1.13	8.8~11.1				
*QWA.caas.7DS*	II, III, A	*Excalibur_c22419_460*	0.00	3.5~4.4	−0.69~–0.82	7.6~10.5	−0.42	0.14	0.01	−0.15
**MIXOLAB DEVELOPMENT TIME (DT)**										
*QDT.caas.1AL*	I, II, III, A	*Glu-A1*	0.02	4.3~5.4	−0.40~–0.54	10.5~11.9	−0.30	−0.03	0.05	−0.03
*QDT.caas.1DL*	I, II, III, A	*BS00018250_51*	0.15	8.5~13.7	−0.44~–0.71	17.7~29.7	−0.21	0.17	0.13	−0.31
*QDT.caas.2AL*	I, II, III, A	*Kukri_c12849_1392*	0.03	2.7~3.7	−0.26~–0.37	6.3~9.8				
*QDT.caas.2DS.1*	I, II	*wsnp_Ex_c12947_20510337*	0.07	2.8~3.0	0.40~0.51	6.5~9.8				
*QDT.caas.2DS.2*	I, II	*wsnp_Ra_rep_c116793_96612614*	2.06	2.9~3.5	−0.40~–0.43	7.5~8.8				
*QDT.caas.2DL*	I, III	*RFL_Contig4790_1091*	0.01	2.8~3.6	−0.28~–0.32	6.4~8.6				
*QDT.caas.4B*	I, III, A	*Kukri_rep_c69273_148*	1.00	2.8~3.4	−0.27~–0.40	7.3~7.9				
*QDT.caas.4BL*	I, II, III, A	*RAC875_rep_c72961_977*	0.04	3.5~4.2	0.32~0.42	7.6~9.5				
**MIXOLAB STABILITY TIME (ST)**										
*QST.caas.1B*	I, II, III, A	*Tdurum_contig43910_1113*	0.01	10.6~22.4	−0.55~–0.83	7.8~15.8				
*QST.caas.1DL*	I, II, III, A	*Glu-D1*	0.08	10.6~12.9	−0.83~–1.16	19.4~26.9	−0.31	0.25	0.26	−0.51
*QST.caas.2BS.1*	I, II, III, A	*Tdurum_contig42636_995*	0.04	2.8~4.2	0.61~0.72	6.0~9.2				
*QST.caas.2BS.2*	I, II, III, A	*Tdurum_contig65756_350*	0.04	3.5~5.5	−0.62~–0.89	7.9~12.0	−0.32	0.02	−0.05	0.03
*QST.caas.3BS*	II, III	*wsnp_Ra_c16264_24873670*	0.08	2.8~3.0	−0.05	6.2~6.4				
*QST.caas.4BS*	I, II, III, A	*Kukri_c34633_69*	0.90	3.0~4.1	−0.49~–0.62	6.8~9.5	−0.32	0.13	0.02	−0.15
*QST.caas.4BL*	I, II, A	*RAC875_rep_c72961_977*	0.04	3.3~4.6	0.57~0.91	7.2~10.0				
*QST.caas.5AS*	I, III, A	*wsnp_Ex_c15046_23216392*	0.30	2.9~3.3	−0.53~–0.62	7.3~7.7				
*QST.caas.6BS*	I, II, A	*BS00077897_51*	0.05	2.6~3.2	−0.51~–0.64	6.5~7.7				
**MIXOLAB C2 (C2)**										
*QC2.caas.1DL*	I, II, III, A	*Glu-D1*	0.08	3.2~8.4	−0.02~–0.03	6.4~18.6				
*QC2.caas.2BS.1*	I, II, A	*Tdurum_contig42636_995*	0.04	2.8~3.5	0.02	6.1~7.8				
*QC2.caas.2BS.2*	I, II, III, A	*Kukri_c14335_2382*	0.02	2.9~6.5	−0.02~–0.03	8.3~15.7	−0.01	0.00	0.00	−0.01
*QC2.caas.4B*	I, II, III, A	*Kukri_rep_c69273_148*	1.00	3.4~3.8	−0.02	8.5~9.2	−0.01	−0.01	0.01	0.00
*QC2.caas.5AS*	I, III, A	*wsnp_Ex_c15046_23216392*	0.30	3.9~5.3	−0.02	8.6~11.0	−0.01	0.00	−0.01	0.01
**MIXOLAB C3 (C3)**										
*QC3.caas.1DS*	II, III	*TA003135-0494*	0.04	2.7~6.8	0.08~0.11	7.4~17.3				
*QC3.caas.3AS.1*	II, III, A	*Excalibur_c48057_110*	0.03	2.7~3.4	−0.06~–0.07	1.7~9.0				
*QC3.caas.3AS.2*	I, II, III, A	*CAP8_c2839_118*	3.05	3.4~4.0	0.05~0.08	8.4~9.9	0.03	−0.01	0.01	0.00
*QC3.caas.3BL*	II, III, A	*Excalibur_c2362_472*	0.02	2.6~4.7	0.07~0.08	9.0~11.0				
*QC3.caas.4BS*	I, II, III, A	*Kukri_c56202_108*	0.02	2.7~4.3	−0.04~–0.06	5.8~8.3	−0.04	0.00	0.00	0.01
*QC3.caas.4BL*	I, II, A	*Excalibur_c20411_127*	0.00	3.2~3.3	−0.05~–0.06	6.7~7.0				
*QC3.caas.5BL.1*	I, III, A	*BS00031339_51*	0.02	4.1~6.4	−0.07~–0.08	8.0~13.0				
*QC3.caas.5BL.2*	II, III, A	*wsnp_Ex_c39535_46808105*	0.00	3.1~5.2	−0.04~–0.07	5.8~11.8	−0.03	0.01	0.03	−0.03
**MIXOLAB C4 (C4)**										
*QC4.caas.2BS*	I, II, A	*RAC875_c56101_368*	0.02	2.7~3.0	−0.05~–0.09	6.3~6.5				
*QC4.caas.4AS*	I, III	*wsnp_Ra_c10536_17322563*	0.02	2.6~3.7	0.06~0.09	5.2~9.0	0.05	0.03	−0.03	−0.01
*QC4.caas.4BL*	I, II, A	*Excalibur_c20411_127*	0.00	3.1~5.8	−0.05~–0.08	6.8~11.7				
*QC4.caas.5BL.1*	I, III, A	*BS00031339_51*	0.02	5.3~7.3	−0.10~–0.13	10.6~15.2	−0.04	−0.03	0.02	0.00
*QC4.caas.5BL.2*	I, III, A	*wsnp_Ex_c16100_24532224*	0.01	2.9~4.2	0.06~0.08	6.0~7.6				
*QC4.caas.6BL*	II, III, A	*BS00022462_51*	0.00	3.5~3.8	0.06~0.13	8.7~8.8				
**MIXOLAB C5 (C5)**										
*QC5.caas.3AS*	II, III	*Excalibur_c25195_1047*	0.02	2.9~4.9	0.12~0.17	7.0~11.7				
*QC5.caas.4AL*	I, III, A	*BobWhite_c8680_993*	0.03	3.0~4.4	−0.17~–0.19	6.9~8.4				
*QC5.caas.5BL*	I, III, A	*BS00031339_51*	0.02	2.9~6.7	−0.09~–0.21	5.5~14.0	−0.13	−0.18	0.10	0.07
*QC5.caas.6DS*	I, III	*RAC875_rep_c85994_258*	1.07	3.0~8.4	−0.17~–0.30	7.8~22.1				
*QC5.caas.7B*	I, III, A	*BS00064367_51*	0.01	2.8~3.1	0.12~0.13	6.0~7.4				
*QC5.caas.7DS*	I, III, A	*GENE-3129_828*	0.74	3.2~6.0	0.13~0.18	7.1~12.1	0.09	0.02	−0.02	0.00

*I: Anyang 2012–2013, II: Anyang 2013–2014, III: Suixi 2013–2014, A: Pooled data for three environments*.

*Add: Additive effect, AbyE: QTL × environment interaction*,

* *: New QTLs for Mixograph and RVA parameters*.

*See footnote of Table 1 for abbreviations*.

### QTLs for Mixograph parameters

MPT, MPV, MPW and MTxW were controlled by 10, 11, 8, and 6 QTLs, respectively (Table 4). *QMPT.caas.1DL*, tightly linked to *GENE-0511_484* with a genetic distance of 0.02 cM, explained the highest phenotypic variation ranging from 37.6 to 52.6% across environments. The QTLs on 1AL and 1BL tightly linked to *Glu-A1* and *H20* with genetic distances of 1.03 and 0.05 cM, respectively, had major effect on MPV and MPW. *QMTxW.caas.1DL, QMTxW.caas.2BS*, and *QMTxW.caas.4B* were identified across three environments, explaining 5.9–12.0% of the phenotypic variation. Alleles with increasing effects for the QTLs influencing Mixograph parameters located on chromosomes 1AL, 1BL, and 1DL were from Gaocheng 8901.

### QTLs for RVA parameters

PV, TV, BD, FV, SB, and PTI were associated with 5, 5, 2, 5, 3, and 9 QTLs, respectively (Table 4). *QPV.caas.6BL.2* tightly linked to *RAC875_c50348_93* at a genetic distance of 5.00 cM showed the highest contribution. The major *QTV.caas.1BL* was 0.02 cM from *Kukri_c7770_176* and explained up to 17.9% of the phenotypic variation. *QBD.caas.7DL* closely linked to *Excalibur_c4508_1007* was detected in all environments, accounting for 10.4–14.0% of the phenotypic variation. *QFV.caas.5BL.2* was 0.01 cM from *BS00031339_51*, explaining up to 13.6% of the phenotypic variation. *QPTI.caas.2AL* and *QPTI.caas.4AS* tightly linked to *Excalibur_c6710_2192* and *RAC875_rep_c106151_123* at genetic distances of 0.00 and 0.03 cM, respectively, were identified in all environments. Several QTLs (*QPV.caas.7BL, QBD.caas.5AS, QBD.caas.7DL, QPTI.caas.1BL, QPTI.caas.3BL*, and *QPTI.caas.7DS*) were strongly affected by QE interaction.

### QTLs for Mixolab parameters

WA, DT, ST, C2, C3, C4, and C5 were conditioned by 13, 8, 9, 5, 8, 6, and 6 QTLs, respectively (Table 4). The marker interval *Ha*-*Excalibur_c49805_63* on chromosome 5DS was associated with WA in all environments. The stable QTL tightly linked to *Glu-D1*, was the most important locus for ST and C2. *QC3.caas.1DS* was 0.04 cM from *TA003135-0494*, explaining 7.4–17.3% of the phenotypic variation. The largest phenotypic variations for C4 and C5 were contributed by *QC4.caas.5BL.1* and *QC5.caas.6DS* closely linked to *BS00031339_51* and *RAC875_rep_c85994_258* at genetic distances of 0.02 and 1.07 cM, respectively. Alleles increasing DT and ST on chromosome 1DL came from Gaocheng 8901 and exhibited strong interaction with environments.

### QTL clusters

**Figure 1:**
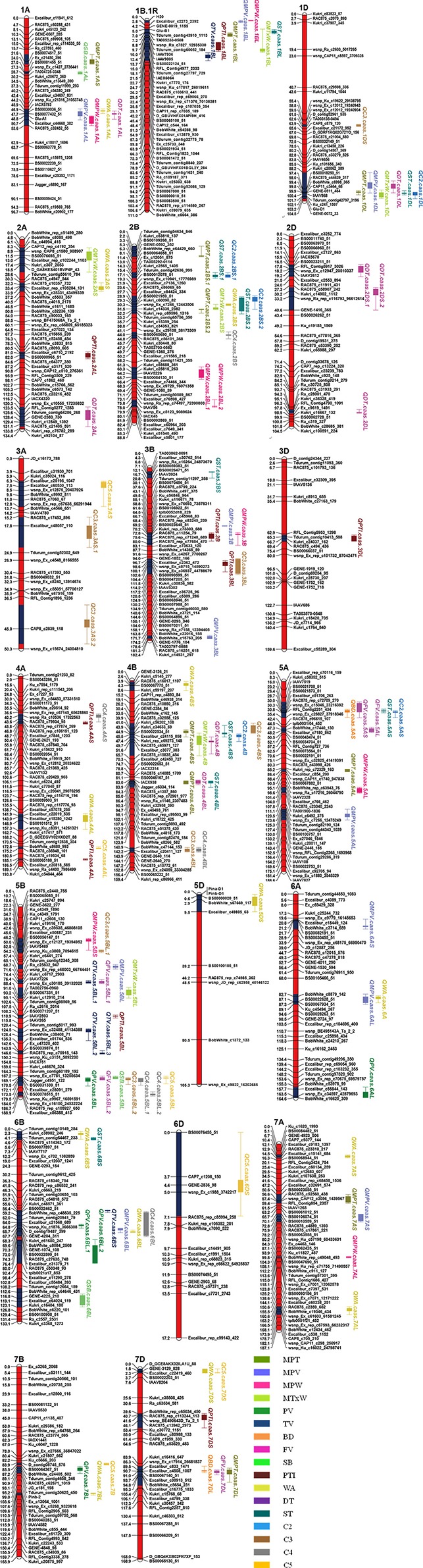
**Genetic maps and QTLs for quality traits identified in the Gaocheng 8901/Zhoumai 16 population from the 90 K iSelect SNP genotyping assay**. Single chromosome was in red and blue region on the chromosome indicated the confidence interval of QTLs detected. QTL names were on the right with different colors for different traits. See footnote to Table 1 for abbreviations.

Some QTLs for different quality parameters were mapped in the same or nearby marker intervals, possibly due to a pleiotropic effect of a single gene or tightly linked genes. Eleven QTL clusters were identified on chromosomes 1AL, 1BL, 1DL, 2BS, 3B, 4B (2), 5AS, 5BL, 6BL, and 7DL based on Map 1 (Table 5, Figure 1). QTL clusters related to dough rheological properties were located on chromosomes 1AL, 1DL, 2BS and 4BL, and those associated with starch pasting properties were identified on chromosome 5BL. The QTL clusters for dough rheological and starch pasting properties were identified on chromosomes 1BL, 3B, 4B, 5AS, 6BL, and 7DL.

**Table 5:** **QTL clusters for quality traits in the Gaocheng 8901/Zhoumai 16 population based on the linkage map from the 90 K iSelect genotyping assay**.

**Chromosome**	**Marker interval**	**Position (cM)**	**Trait**
1AL	*wsnp_Ku_c21316_31053745~RAC875_c32452_55*	45.3~53.6	MPV, MPW, WA, DT
1BL	*Glu-B1~Kukri_c7770_176*	5.0~22.7	MPT, MTxW, TV, PTI, ST
1DL	*Kukri_c20062_389~Glu-D1*	88.6~103.2	MPT, MPV, MTxW, DT, ST, C2
2BS	*Ku_c69635_2786~RAC875_c3067_1830*	24.9~34.2	MPT, MTxW, WA, ST, C2
3B	*BS00072151_51~GENE-1617_131*	61.3~81.9	MPV, MPW, PTI
4B	*Excalibur_c16698_123~RAC875_c15835_454*	35.8~63.1	MPT, MTxW, DT, ST, C2, C3
4BL	*IACX938~Kukri_c32958_390*	83.5~89.7	MPT, DT, ST
5AS	*Kukri_c18268_79~Kukri_c60091_331*	20.1~35.8	BD, FV, ST, C2
5BL	*Kukri_c2735_626~wsnp_Ku_c9967_16591591*	167.9~177.8	PV, FV, SB, C3, C4, C5
6BL	*Kukri_c38732_246~wsnp_RFL_Contig2747_2479869*	55.1~73.7	MPV, PV, TV, WA, C4
7DL	*wsnp_Ex_c17914_26681837~Kukri_c15768_68*	86.3~103.5	MPT, BD, FV

*See footnote of Table 1 for abbreviations*.

### Important QTLs and QTL clusters based on the high-density linkage map with 90 and 660 K chips

*QC3.caas.3AS.2*, detected in all environments, explained 8.4–9.9% of the phenotypic variation. However, this region represented a gap of 10.0 cM in the linkage map from the 90 K assay. Additional six markers from the 660 K chip were newly mapped to this region and delineated this QTL to a 2.0 cM interval flanked by markers *CAP8_c2839_118* and *AX-110915310* (Figure S2). The distance of the closest marker to the LOD contour peak of *QMPV.caas.5AL* was shortened from 1.1 to 0.7 cM by the additional marker from the 660 K assay. An additional two markers from the 660 K assay were mapped to the QTL cluster on chromosome 4B in the region of 47.4-49.7 cM, and the interval of *QMTxW.caas.4B, QDT.caas.4B* and *QST.caas.4BS* was reduced from 2.3 to 1.6 cM, flanked by markers *AX-109563308* and *AX-111689026*. An additional two markers from the 660 K assay were newly mapped to the QTL cluster on chromosome 5AS in the region of 30.8–35.4 cM, and the interval for *QFV.caas.5AS* was shortened from 4.6 to 0.3 cM, flanked by markers *RFL_Contig2251_434* and *AX-108730091*.

## Discussion

### Phenotypic evaluation

Mixograph and RVA are most commonly used to evaluate the dough rheological characteristics and starch pasting properties in wheat breeding, respectively. Recently, a new device Mixolab has been introduced to assess dough rheological characteristics, starch pasting properties and flour enzyme activity in one test sample, reducing labor requirements. The instrument also provide information on effects of different ingredients (Bonet et al., 2006; Rosell et al., 2007; Marco and Rosell, 2008), indicating that it could be used in areas of the bakery products industry. Since, it is a relatively new device, the correlations between Mixolab parameters and other traditional instruments such as Mixograph and RVA have been not completely established. The significant correlations between MPT and Mixolab parameters for dough properties such as DT (*r* = 0.80), ST (*r* = 0.85), and C2 (*r* = 0.60) were found in the present study. FV also exhibited significant correlations with C3 (*r* = 0.31), C4 (*r* = 0.44), and C5 (*r* = 0.38). These results suggested that wheat quality can be effectively assessed by Mixolab parameters. However, it needs about 50-g flour in one Mixolab test, whereas only 10 and 3.5-g of flour were needed in one Mixograph and RVA analysis, respectively. As a result, Mixograph and RVA are more suitable for analyzing early generational material in wheat quality breeding while Mixolab could be used in bakery factories.

Although the present study showed genotype, environment and QE interaction had significant effects on all traits except MPW and PTI, the broad-sense heritabilities assessed for all traits ranged from 0.56 to 0.94, suggesting that genotype had the largest contribution to source of variation, in agreement with Patil et al. (2009).

### High-density linkage map

Linkage maps play a crucial role in identifying QTL, cloning genes, MAS and genome structure analysis (Maccaferri et al., 2014). The QTL mapping was based on the genotyping data with the 90 K array. Because a low coverage of SNP markers on some chromosomal regions, several QTLs were mapped in larger marker intervals. Thus, the 660 K SNP array was used to genotype two parents and 59 RILs randomly chosen from the population to saturate the chromosomal regions with larger marker intervals. In the present study, we firstly obtained a high-density linkage map based on the 90 and 660 K iSelect SNP assays, including 46,961 polymorphic SNP markers. Of these markers, 6596 (1046 from the 90 K SNP assay and 5550 from the 660 K SNP assay) were newly anchored to the bread wheat linkage map (Table S4), by comparing with the 90 and 660 K consensus maps (Wang et al., 2014; Prof. Jizeng Jia, pers. comm.). The average density of the map was 0.09 cM/marker, indicating a higher marker-density than developed previously with DArT (Echeverry-Solarte et al., 2015) or SNP from the 90 K assay (Gao et al., 2015). The marker-densities in regions surrounding important QTLs (*QC3.caas.3AS.2* and *QMPV.caas.5AL*) and QTL clusters (4B, 5AS, 5BL, and 7DL) were significantly increased by the high-density linkage map (Figure S2); consequently increasing the resolution of QTL mapping. It is interesting that the original marker orders of these regions, except for chromosome 7DL from the 90 K SNP assay, are unchanged in the high-density linkage map, indicating that the regions are conserved. Although a good coverage of the genomes was obtained, polymorphism in the D genome was still relatively low. In summary, this high-density linkage map is valuable for fine mapping, candidate gene discovery and MAS in wheat breeding.

### Comparison with previous studies

It was proven that HMW-GSs showed larger contribution to dough properties (Branlard et al., 2001; He et al., 2005; Liu et al., 2005). In the present study, the major effect QTLs for Mixograph parameters on chromosomes 1AL, 1BL, and 1DL should be conferred by HMW-GSs flanked by *BS00030036_51* and *Excalibur_c44668_382, Glu-B1* and *wsnp_Ra_c7527_12935330, BS00018250*, and *Glu-D1*, respectively, in agreement with previous reports (Nelson et al., 2006; Mann et al., 2009).

The stable QTL on chromosome 2BS with Gaocheng 8901 allele increased MPT (*QMPT.caas.2BS.1*) and MTxW(*QMTxW.caas.2BS*). Zhang et al. (2009) indicated that an appropriate ratio of quantity of glutenin to gliadin had larger contribution to dough Mixograph properties. This QTL may be corresponding to the region related to the quantity of gluten or gliadin protein fractions in grain protein using a genome-wide association study of a bread wheat world core collection (Plessis et al., 2013). *QMPV.caas.3BL*, with allele from Zhoumai 16 increasing MPV, is in a similar position on chromosome 3BL that influenced glutenin macropolymer content, Zeleny sedimentation volume and grain protein content (Sun et al., 2008). This coincides with previous study that there was significant correlation between protein content and MPV (Tronsmo et al., 2003).

Gaocheng 8901 (HI = 66) with alleles *Pina-D1b*/*Pinb-D1a*/*Pinb-2v2* showed slightly harder than Zhoumai 16 (HI = 53) with *Pina-D1a*/*Pinb-D1b*/*Pinb-2v3*. Grain hardness mainly affected by *Ha* locus on chromosome 5DS exhibited significant influence on milling quality. It has been reported that hard wheat has much more the amount of damaged starch than soft wheat (Barak et al., 2012). The association analysis of genotypic and phenotypic data using *T*-test indicated that both *Pina* and *Pinb* conferred significant effect on WA (*P* < 0.01). Therefore, QTL for WA on chromosome 5DS was contributed by both *Pina* and *Pinb* genes at *Ha* locus, in agreement with Ma et al. (2007).

One stable *QMPT.caas.5AL* positioned at 87 cM on chromosome 5AL was not previously reported. A stable QTL *QMPT.caas.7DL* at 93 cM on chromosome 7DL is different from one reported by Tsilo et al. (2011) at position of 12 cM on chromosome 7DS. *QMPV.caas.3B, QMPV.caas.3BL* and *QMPV.caas.6BL* were not previously reported. *QMPV.caas.5AL* at 128 cM is different from one reported by Li et al. (2012) on chromosome 5AS. *QMPW.caas.5BS* and *QMPW.caas.7AL* are reported for the first time. *QMPW.caas.2BL.1* and *QMPW.caas.2BL.2*, are different from a QTL on chromosome 2BS reported by McCartney et al. (2006). *QMTxW.caas.2BS* and *QMTxW.caas.5BL* are new QTLs.

Functional genes, transporters and transcription factors associated with starch metabolism were summarized by Singh et al. (2015). The sequences of these genes were used as queries against the T3 marker database (Zhai et al., 2016; http://triticeaetoolbox.org/wheat/viroblast/viroblast.php) to identify SNP markers corresponding to the original genes, and then the genetic map constructed in the present study was inspected for the presence of the same markers. *Kukri_rep_c101946_496* derived from an isoamylase 2 gene, was mapped on chromosome 1AL at a distance of 2.45 cM from the LOD contour peak marker for *QSB.caas.1AL*. *Wsnp_Ex_rep_c66900_65313836* derived from an isoamylase 3 gene, was mapped on chromosome 5AL at a distance of 9.50 cM from the LOD contour peak marker of *QFV.caas.5AL* (Table S5). The QTL on chromosome 7D is not a *Wx* gene since both parents were wild type at all three *Wx* loci.

*QTV.caas.1BL, QTV.caas.5BL.1, QTV.caas.5BL.2*, and *QTV.caas.5BL.3* are reported for the first time. *QTV.caas.6BS* positioned at 56 cM on chromosome 6BS is different from one reported on chromosome 6BL by Sun et al. (2008). *QBD.caas.5AS* and *QBD.caas.7DL* are new QTLs. *QFV.caas.5AS, QFV.caas.5AL*, and *QFV.caas.7DL* are also new. *QSB.caas.6BL* positioned at 116 cM on chromosome 6BL was mapped in a similar position to a QTL reported by Sun et al. (2008). *QSB.caas.1AL* and *QSB.caas.5BL* are new QTLs. *QPTI.caas.1BL, QPTI.caas.2AL, QPTI.caas.3B, QPTI.caas.3BL*, and *QPTI.caas.5BL* are reported for the first time.

Arabinoxylans are separated into water-extractable (WE-AX) and water-unextractable (WU-AX) based on the solubility. It is widely accepted that WE-AX showed positive influence on dough properties because it could construct networks with protein and starch by hydrogen bonding (Yang et al., 2016). Yang et al. (2016) discovered that the QTL cluster for WE-AX and FV in the same region of chromosome 5BL. In the present study, the QTL cluster flanked by *Kukri_c2735_626* and *wsnp_Ku_c9967_16591591* on chromosome 5BL, which is related to PV (*QPV.caas.5BL*), FV(*QFV.caas.5BL.2*), SB (Q*SB.caas.5BL*), C3 (*QC3.caas.5BL.2*), C4 (*QC4.caas.5BL.1*), and C5 (*QC5.caas.5BL*) may represent the region affecting WE-AX.

Although 119 additive QTLs were detected for dough rheological and starch pasting properties, 50 of them were grouped into 11 clusters. QTL clusters for dough strength on chromosomes 1AL, 1BL, and 1DL detected in the present study were associated with HMW-GSs, consistent with previous reports (Huang et al., 2006; Deng et al., 2013). The interval 24.9–34.2 cM on chromosome 2BS is a QTL cluster impacting MPT, MTxW, WA, ST and C2, which coincides with their significant phenotypic correlations. A similar co-localized region for dough development time, stability and softening was also located on chromosome 2B (Maphosa et al., 2015). A QTL cluster for MPV, MPW and PTI between *BS00072151_51* and *GENE-1617_131*, positioned in the interval of 61.3–81.9 cM on chromosome 3B, is likely to be in the same region for MPT, MPI, and MTxW reported by Deng et al. (2013). Another QTL cluster for MPT, MTxW, DT, ST, C2, and C3 identified in the interval 35.8–63.1 cM on chromosome 4B, is likely the same as a QTL cluster for Mixograph midline peak time, midline tail width, envelope peak energy and envelope peak time (Patil et al., 2009). The interval 83.5–89.7 cM on chromosome 4BL is a QTL cluster influencing MPT, DT and ST, in agreement with their phenotypic correlations. This QTL cluster is reported for the first time.

The QTL cluster for BD, FV, ST, and C2 between *Kukri_c18268_79* and *Kukri_c60091_331* positioned in the interval 20.1–35.8 cM on chromosome 5AS is reported for the first time. Another QTL cluster for PV, FV, SB, C3, C4, and C5 in the interval 167.9–177.8 cM on chromosome 5BL is also new. The QTL cluster for MPV, PV, TV, WA, and C4 was in the interval 55.1–73.7 cM on chromosome 6BL, is in a similar pleiotropic region for Farinorgraph dough stability, PV, TV, FV, and PTI between *Xgwm644* and *Xgwm608b* reported previously (Sun et al., 2008). The QTL cluster for MPT, BD, and FV in interval 86.3–103.5 cM on chromosome 7DL is new. The presence of QTL cluster may be attributed to either pleiotropic effects of a single QTL or a few loci closely linked. Most of traits involved in a QTL cluster were significantly correlated (Table 3).

### Potential implication in wheat breeding

KASP is used to detect InDels or SNPs. It is suitable for genotyping several SNP markers for a number of samples. SNP markers closely linked to traits identified by genome-wide association study or QTL mapping will be successfully converted into KASP assay for marker-assisted breeding in the future. In this study, the stable QTLs with larger contribution such as *QMPT.caas.1BL, QMPT.caas.1DL, QMPV.caas.1AL, QMPV.caas.1BL, QMPV.caas.5AL, QMPV.caas.6BL*, and *QMTxW.caas.4B* can be used for MAS of dough rheological characteristics and *QPV.caas.6BL.2, QSB.caas.5BL, QPTI.caas.2AL, QPTI.caas.4AS* are suitable for MAS in improvement of starch pasting properties. In addition, 11 QTL clusters and closely linked markers are also useful for improving wheat processing quality.

The development of a high density linkage map with the wheat 90 K array and comparative genomics provide a powerful tool in searching for potential candidate genes in wheat. Bioinformatics analysis of the mapped SNP markers in the important QTL regions for dough rheological and starch pasting properties identified eight candidate genes. Of them, four genes were involved in biosynthesis of amino acids, two related to starch and sucrose metabolism, and two associated with fatty acid biosynthesis (Table S6). However, since a number of biological processes are associated with these candidate genes, more detailed experimental analyses will be needed to confirm their roles in dough strength and starch pasting properties.

Although there are many reports on QTL for quality traits in wheat (Huang et al., 2006; McCartney et al., 2006; Sun et al., 2008; Patil et al., 2009; Zhao et al., 2009; Tsilo et al., 2011; Li et al., 2012; Deng et al., 2013, 2015; Zheng et al., 2013; Prashant et al., 2015), few genes are identified in single QTL analysis. The main reason is that SSR markers previously used in QTL analysis have insufficient density and are often in non-coding regions. The 90 and 660 K iSelect SNP genotyping assays are developed from transcriptome and genome sequencing, respectively (Wang et al., 2014; Prof. Jizeng Jia, pers. comm.), and therefore provide the better approach in searching for candidate genes.

With rapid development of next-generation sequencing, genomic sequence of many species such as rice (*Oryza sativa* L.) (Goff et al., 2002), brachypodium (*Brachypodium distachyum* L.), (The International Brachypodium Initiative, 2010), wheat (*Triticum aestivum* L.) (Brenchley et al., 2012), barley (*Hordeum vulgare* L.) (The International Barley Genome Sequencing Consortium, 2012) were released on web, which provides possibility for comparative genomics analysis. Syntenic relationships among grass species are served as a tool for fine mapping and predicting gene function (Sorrells et al., 2003; Ouyang et al., 2014). As shown in Figure S3, collinearity analysis on QTL confidence interval indicated that the collinearity between wheat and barley was better than Brachypodium and rice, and the homologies among chromosomes were consistent with previous reports (Sorrells et al., 2003; The International Brachypodium Initiative, 2010). Several QTLs (*QPTI.caas.3B, QPTI.caas.3BL, QC3.caas.3BL, QPTI.caas.3DL, QPTI.caas.3B, QBD.caas.5AS, QC2.caas.5AS, QFV.caas.5AL, QMPW.caas.5AL, QC3.caas.5BL.1, QWA.caas.6A, QMPV.caas.6AL*, and *QWA.caas.7AS*) showed the best conservation of gene order among these species, suggesting that these regions may facilitate further fine mapping and discovery of candidate genes.

## Conclusion

The present study indicates that MPT, MPV, PV, SB, and PTI are good parameters for the evaluation of processing quality in wheat breeding due to higher broad-sense heritabilities. A high-density linkage map was constructed in the Gaocheng 8901/Zhoumai 16 population with the 90 and 660 K SNP arrays; it provided a powerful tool to identify QTLs/genes for important quality traits and candidate genes. Eleven QTL clusters for dough strength and starch pasting properties were discovered. The SNP markers tightly linked to *QMPT. caas.1BL, QMPT.caas.1DL, QMPV.caas.1AL, QMPV.caas.1BL, QMPV.caas.5AL, QMPV.caas.6BL, QMTxW.caas.4B, QPV.caas.6BL.2, QSB.caas.5BL, QPTI.caas.2AL, QPTI.caas.4AS*, and QTL clusters can be used in MAS for improvement of wheat processing quality.

## Ethics statement

We declare that these experiments comply with the ethical standards in China.

## Author contributions

HJ carried out the experimental and wrote the paper. WW, JL, YZ, and JY participated in field trials. SZ contributed to flour milling. ZL, XX, and ZH designed the experiment and assisted in writing the paper.

### Conflict of interest statement

The authors declare that the research was conducted in the absence of any commercial or financial relationships that could be construed as a potential conflict of interest.

## Supplementary material

Figure S1 **Frequency distribution of all quality traits in the Gaocheng 8901/Zhoumai 16 population. (A)** Mixograph midline peak time, **(B)** Mixograph midline peak value, **(C)** Mixograph midline peak width, **(D)** Mixograph midline 8 min band width, **(E)** RVA peak viscosity, **(F)** RVA trough viscosity, **(G)** RVA breakdown, **(H)** RVA final viscosity, **(I)** RVA setback, **(J)** RVA peak time, **(K)** Mixolab water absorption, **(L)** Mixolab development time, **(M)** Mixolab stability time, **(N)** Mixolab protein weakening torque, **(O)** Mixolab starch gelatinization peak torque, **(P)** Mixolab starch gelatinization trough torque, **(Q)** Mixolab starch gelatinization final torque.

Figure S2 **The marker-densities in regions surrounding important QTLs (***QC3.caas.3AS.2*** and ***QMPV.caas.5AL***) and QTL clusters (4B, 5AS, 5BL, and 7DL) were significantly increased by the high-density linkage map**. See footnote of Table 1 for abbreviations.

Figure S3 **Comparative genomics analysis of QTLs detected in wheat (***Triticum aestivum*** L.) with barley (***Hordeum vulgare*** L.), brachypodium (***Brachypodium distachyum*** L.), and rice (***Oryza sativa*** L.)**. See footnote of Table 1 for abbreviations.

Table S1 **PCR primers for gene-specific markers used for genotyping the parents and RILs**.

Table S2 **All SNP markers mapped in the Gaocheng 8901/Zhoumai 16 population**.

Table S3 **Features of the high-density linkage map summarized by chromosome**.

Table S4 **Detailed information for newly mapped markers identified from the 90 and 660 K SNP chips**.

Table S5 **SNP markers retrieved from functional genes associated with starch pasting properties**.

Table S6 **Putative functions of genes corresponding to mapped SNP markers in the important QTL regions based on the linkage maps from the 90 and 660 K iSelect genotyping assays**.

## Abbreviations

BD: RVA breakdown
C2: Mixolab protein weakening torque
C3: Mixolab starch gelatinization peak torque
C4: Mixolab starch gelatinization trough torque
C5: Mixolab starch gelatinization final torque
DT: Mixolab development time
FV: RVA final viscosity
GBSS: Granule-bound starch synthase
HMW-GS: High-molecular-weight glutenin subunit
KASP: Kompetitive Allele Specific PCR
LMW-GS: Low-molecular-weight glutenin subunit
LOD: Logarithm of odds
MAS: Marker-assisted selection
MPT: Mixograph midline peak time
MPV: Mixograph midline peak value
MPW: Mixograph midline peak width
MTxW: Mixograph midline 8 min band width
PTI: RVA peak time
PV: RVA peak viscosity
QTL: Quantitative trait locus
RIL: Recombinant inbred line
RVA: Rapid Visco-Analyzer
SB: RVA setback
SNP: Single nucleotide polymorphism
SSR: Simple sequence repeat
ST: Mixolab stability time
TV: RVA trough viscosity
WA: Mixolab water absorption
WE-AX: Water-extractable arabinoxylans
WU-AX: Water-unextractable arabinoxylans.
