# Supplementary figures and images for: Genome-Wide QTL Mapping for Wheat Processing Quality Parameters in a Gaocheng 8901/Zhoumai 16 Recombinant Inbred Line Population
Source: Front Plant Sci. 2016 Jul 19;7:1032. doi: 10.3389/fpls.2016.01032 (PMC4949415; doi:10.3389/fpls.2016.01032)

**a**

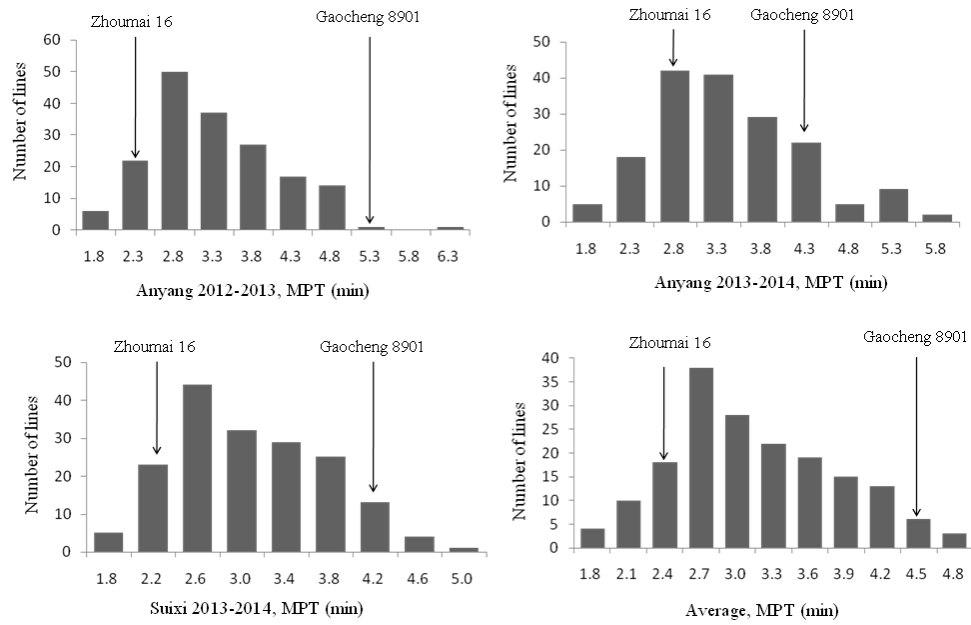

**b**

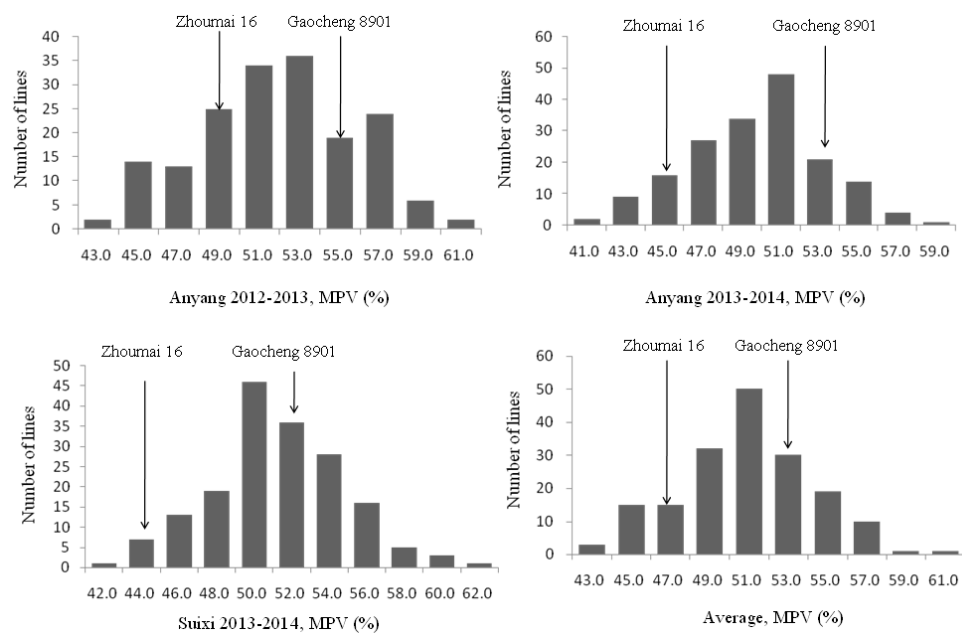

c

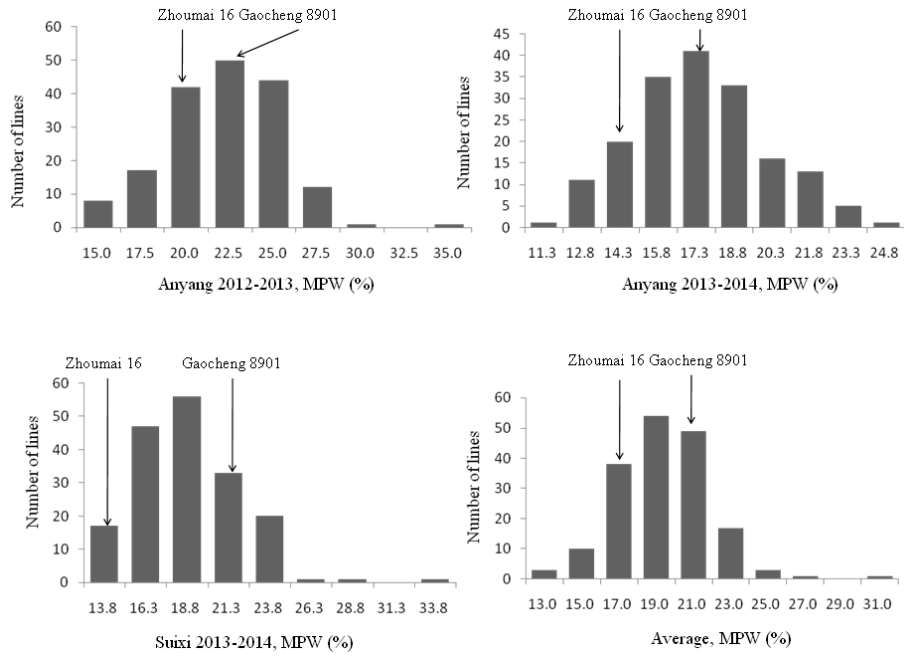

d

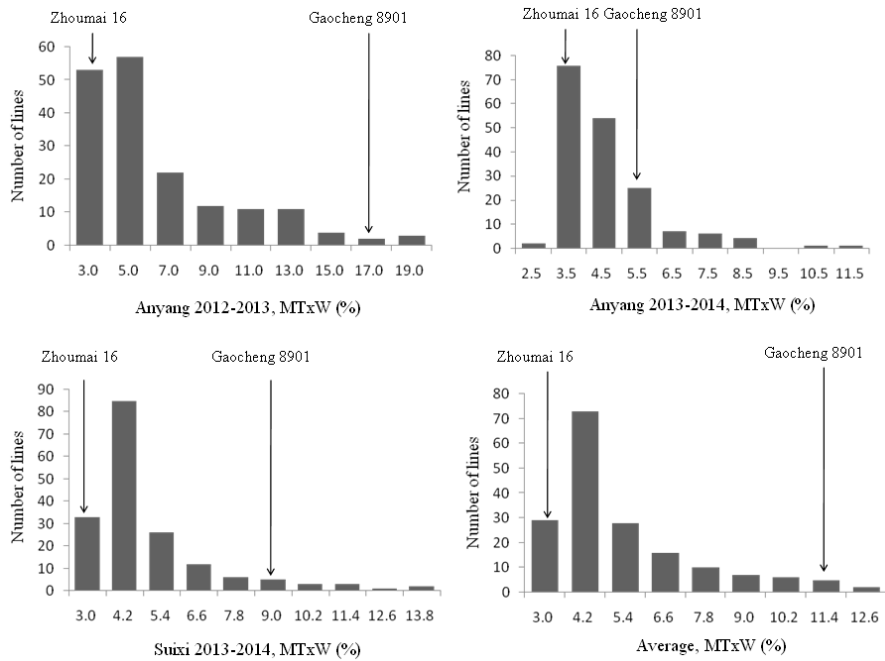

e

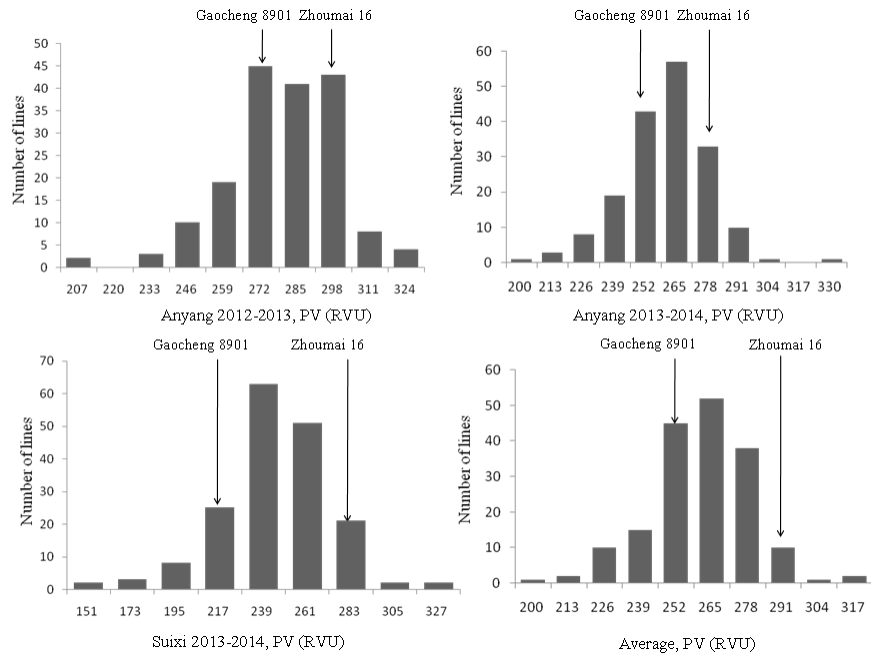

f

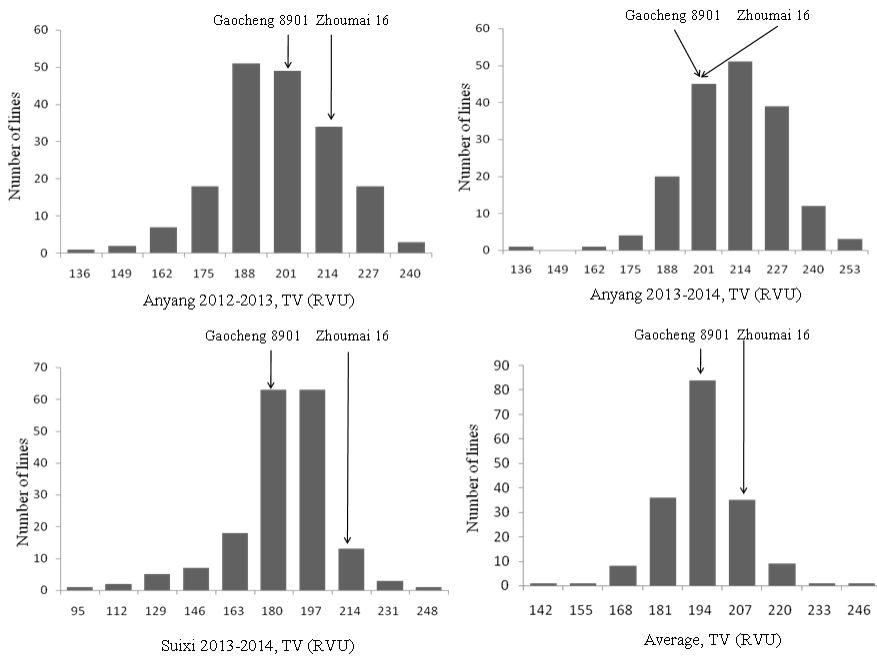

g

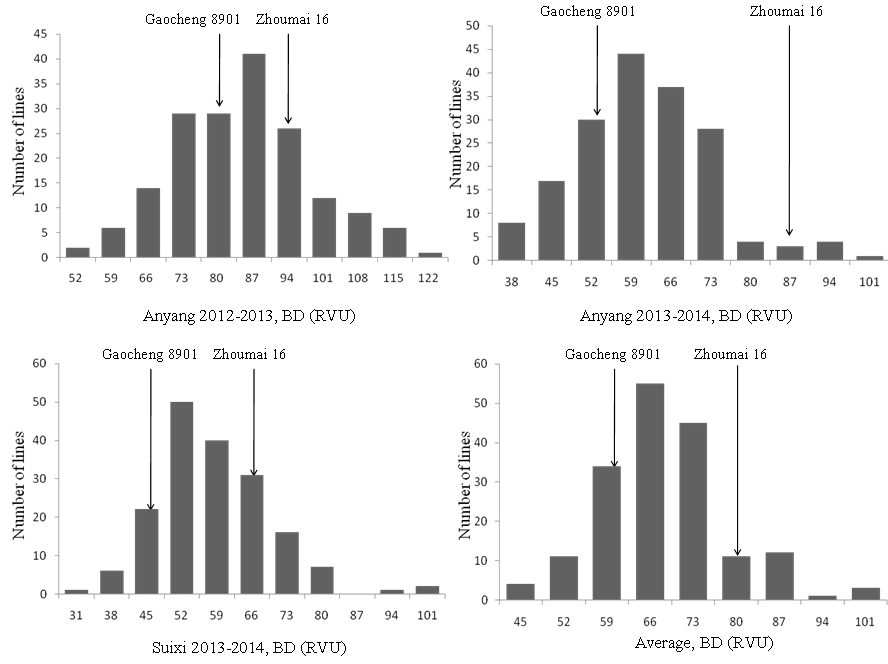

h

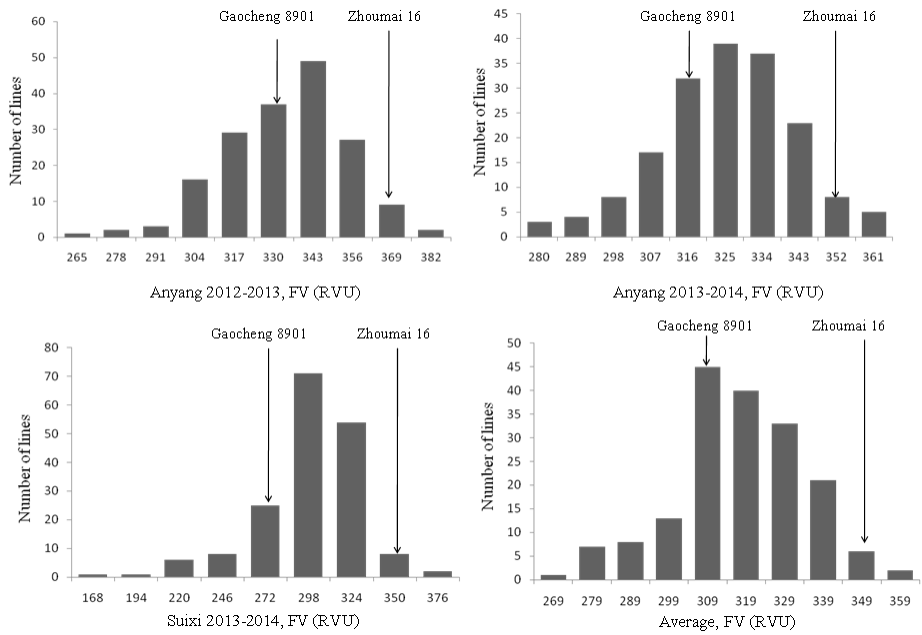

i

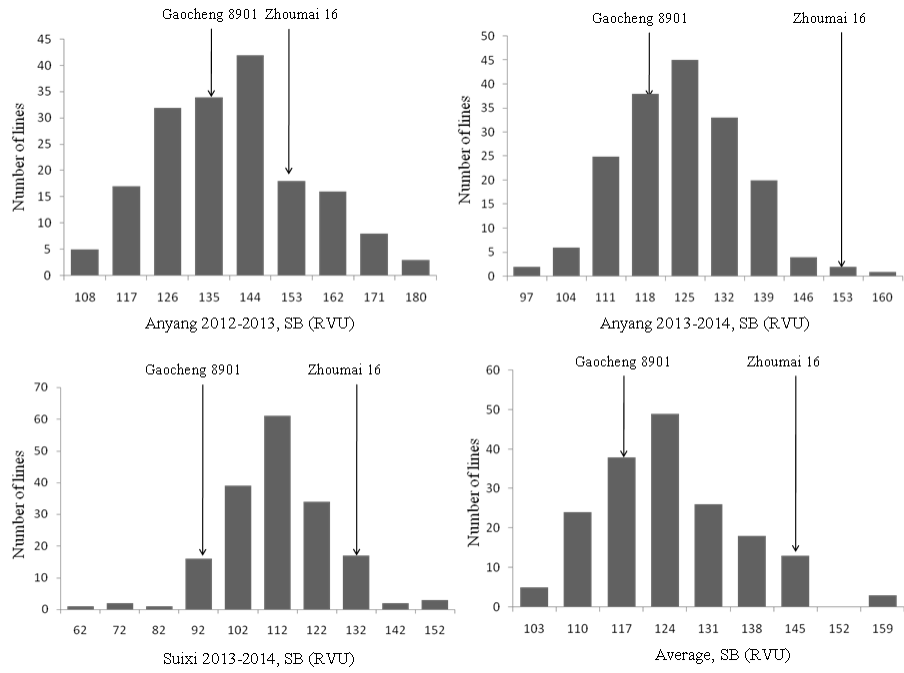

j

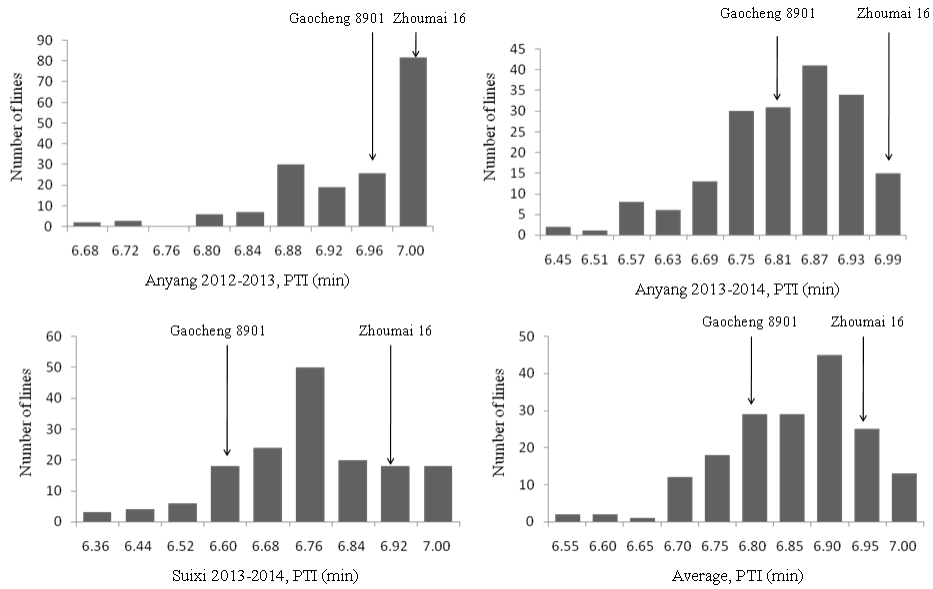

k

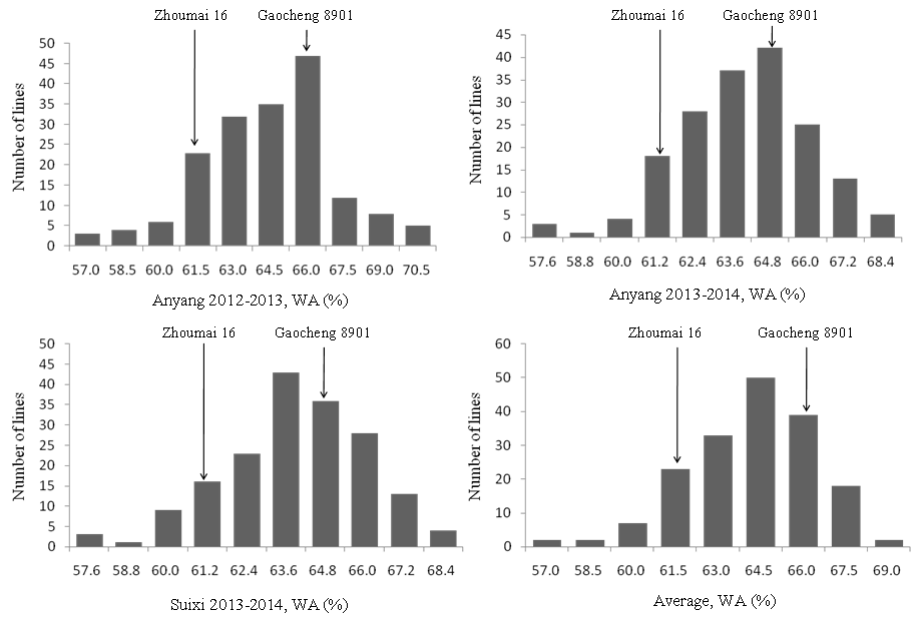

l

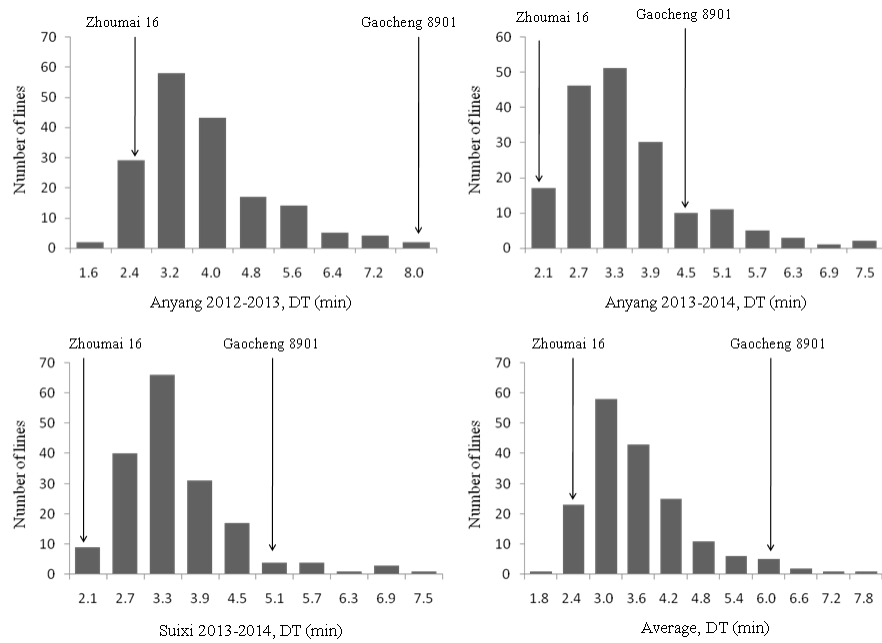

m

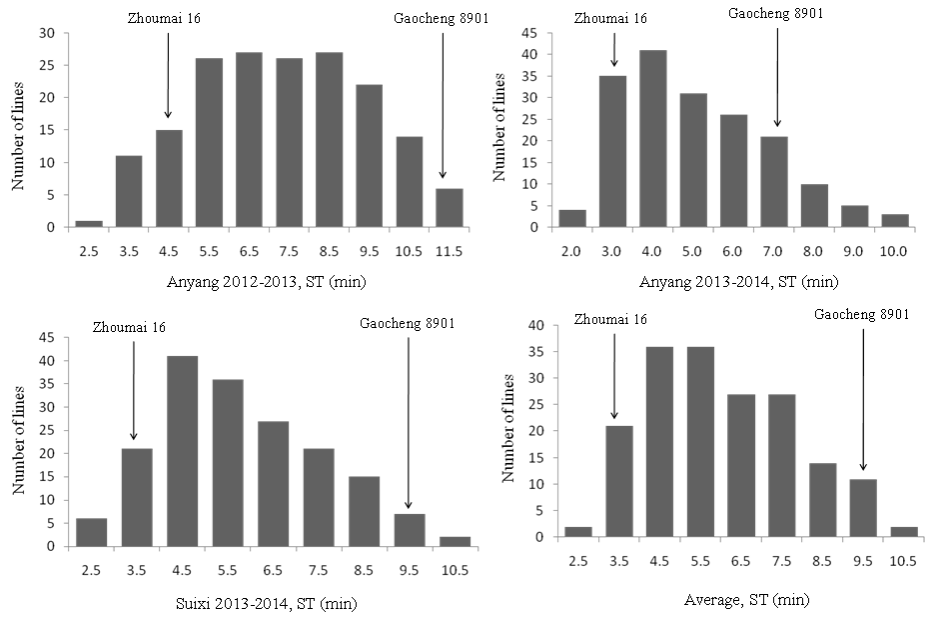

n

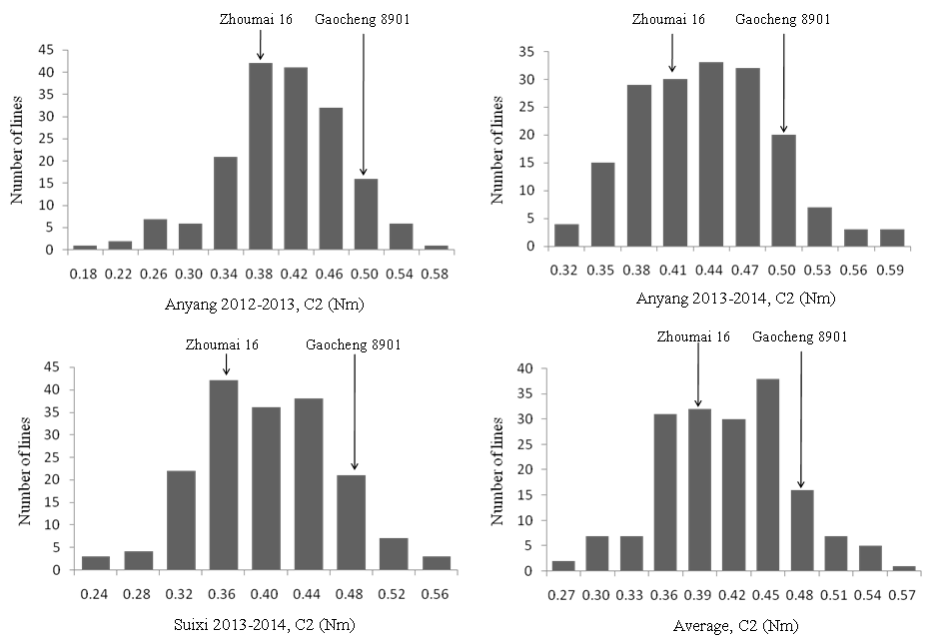

0

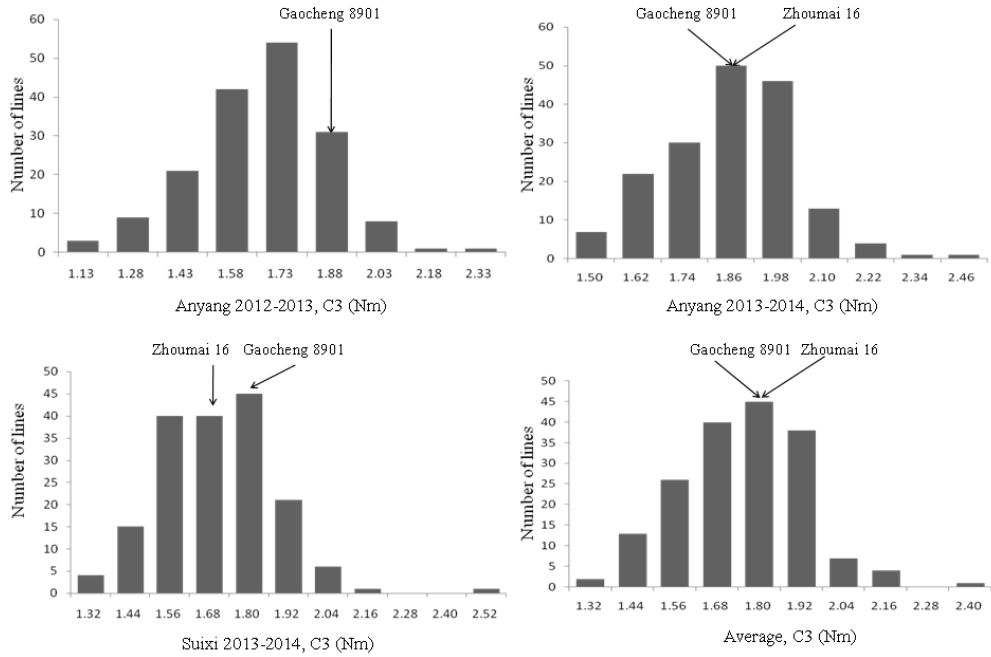

p

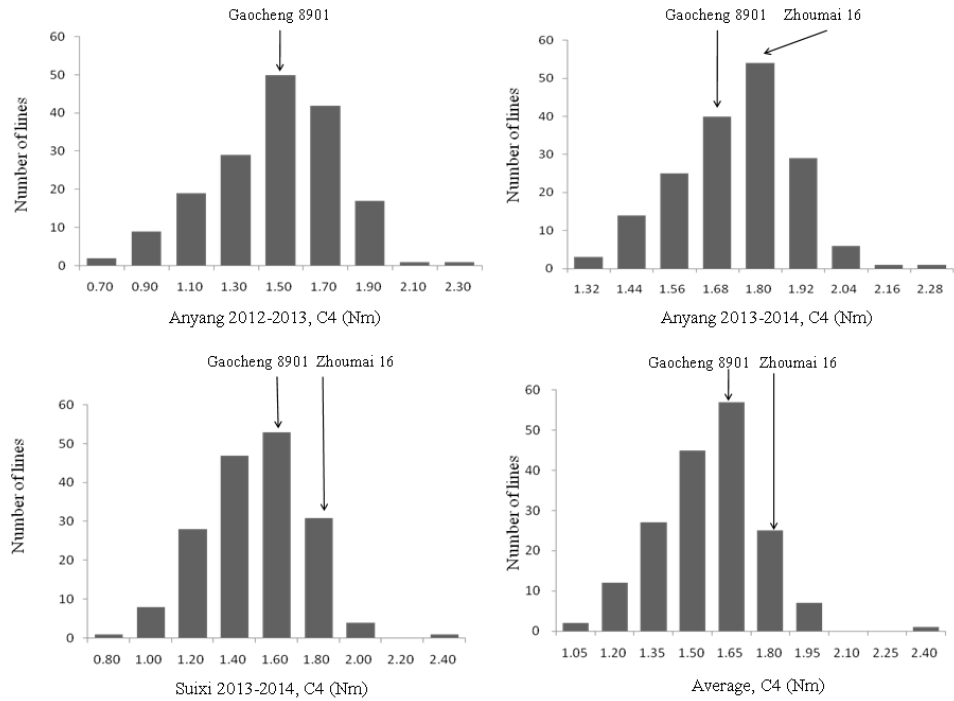

q

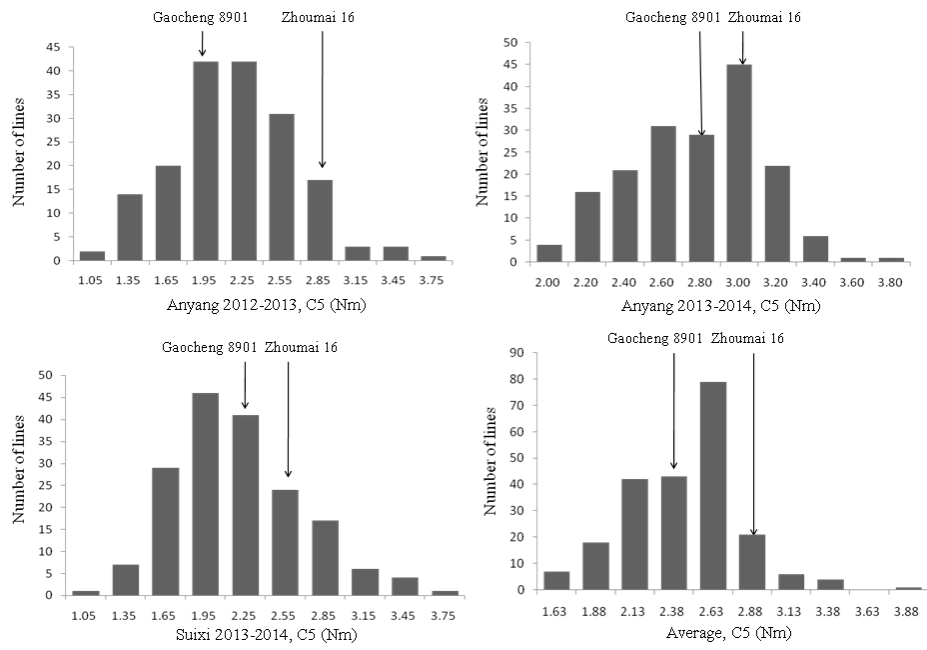

Figure S1

Supplement: Figure S1 — Frequency distribution of all quality traits in the Gaocheng 8901/Zhoumai 16 population. (A) Mixograph midline peak time, (B) Mixograph midline peak value, (C) Mixograph midline peak width, (D) Mixograph midline 8 min band width, (E) RVA peak viscosity, (F) RVA trough viscosity, (G) RVA breakdown, (H) RVA final viscosity, (I) RVA setback, (J) RVA peak time, (K) Mixolab water absorption, (L) Mixolab development time, (M) Mixolab stability time, (N) Mixolab protein weakening torque, (O) Mixolab starch gelatinization peak torque, (P) Mixolab starch gelatinization trough torque, (Q) Mixolab starch gelatinization final torque. [file Image1.PDF]

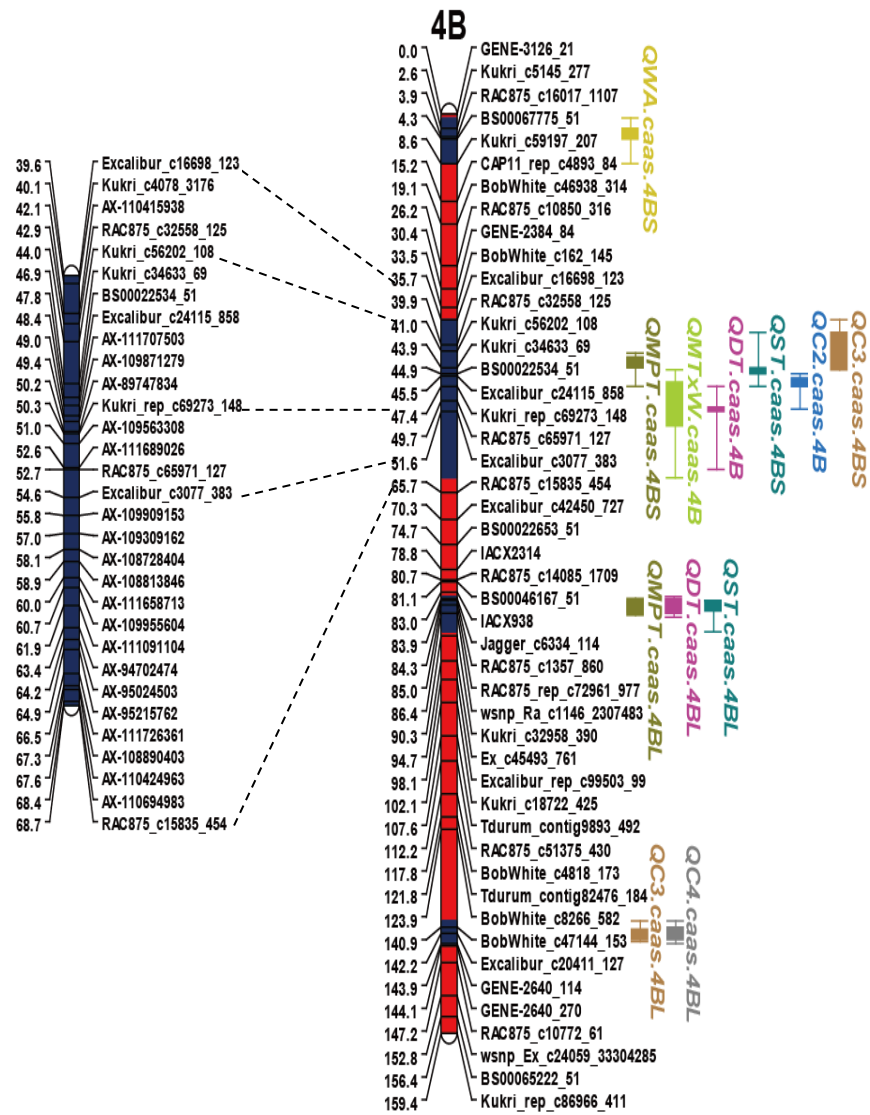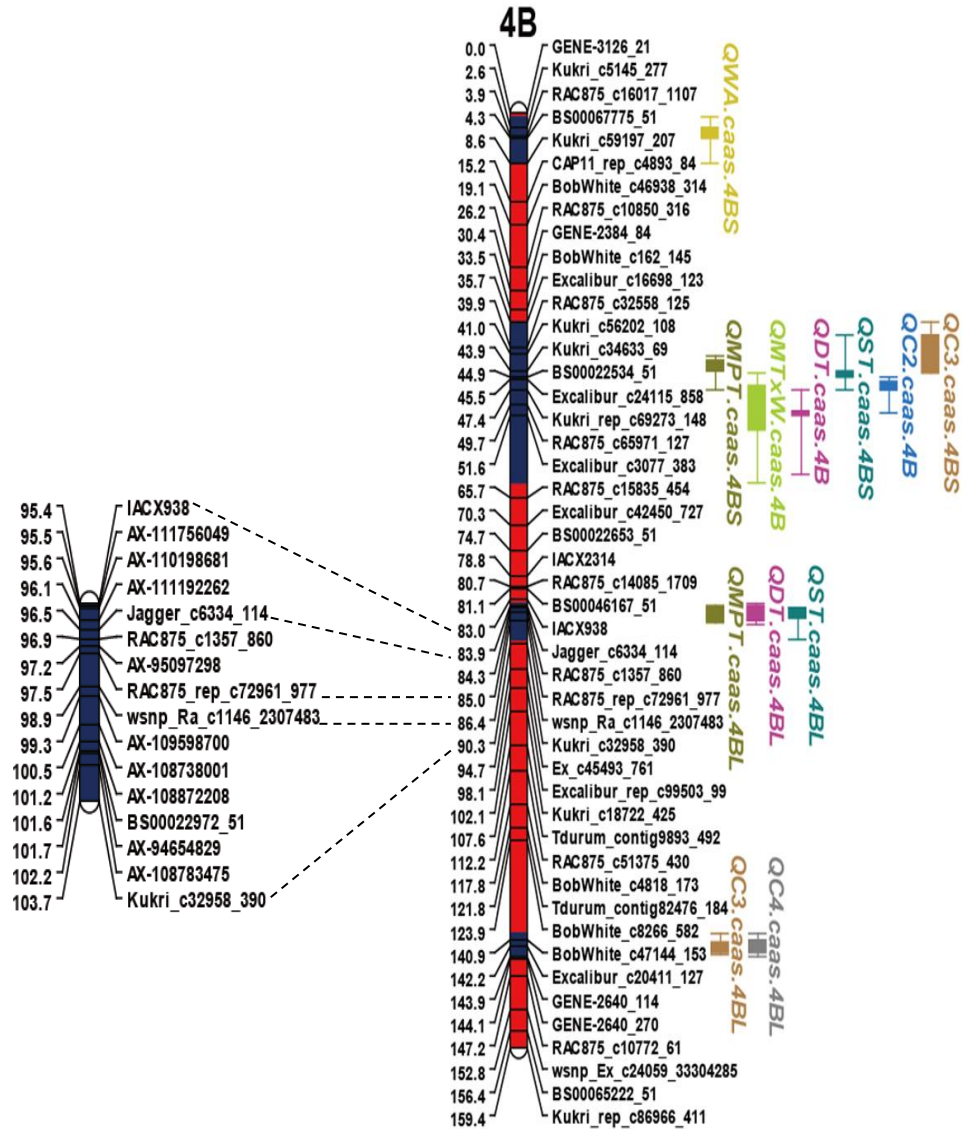

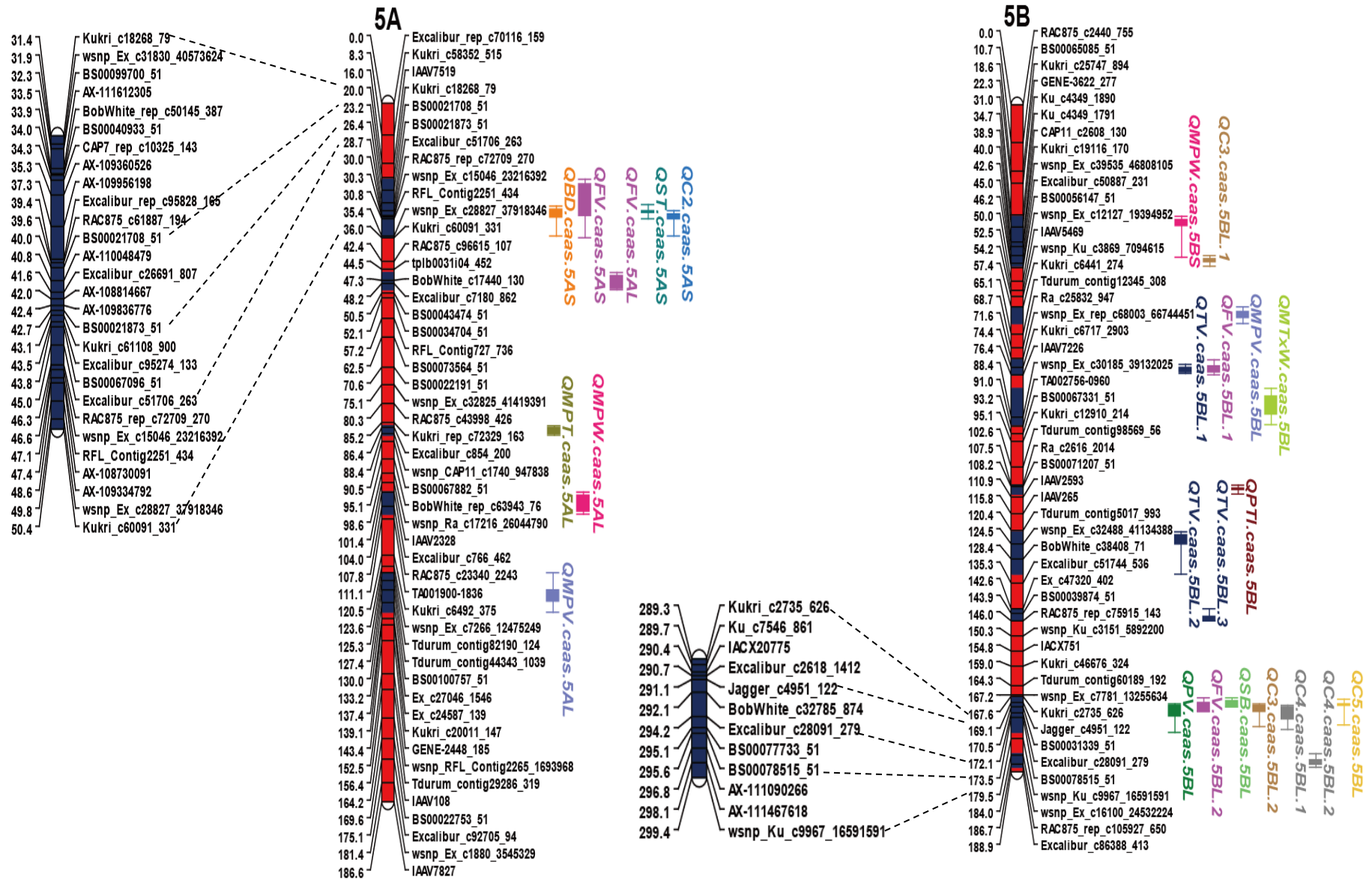

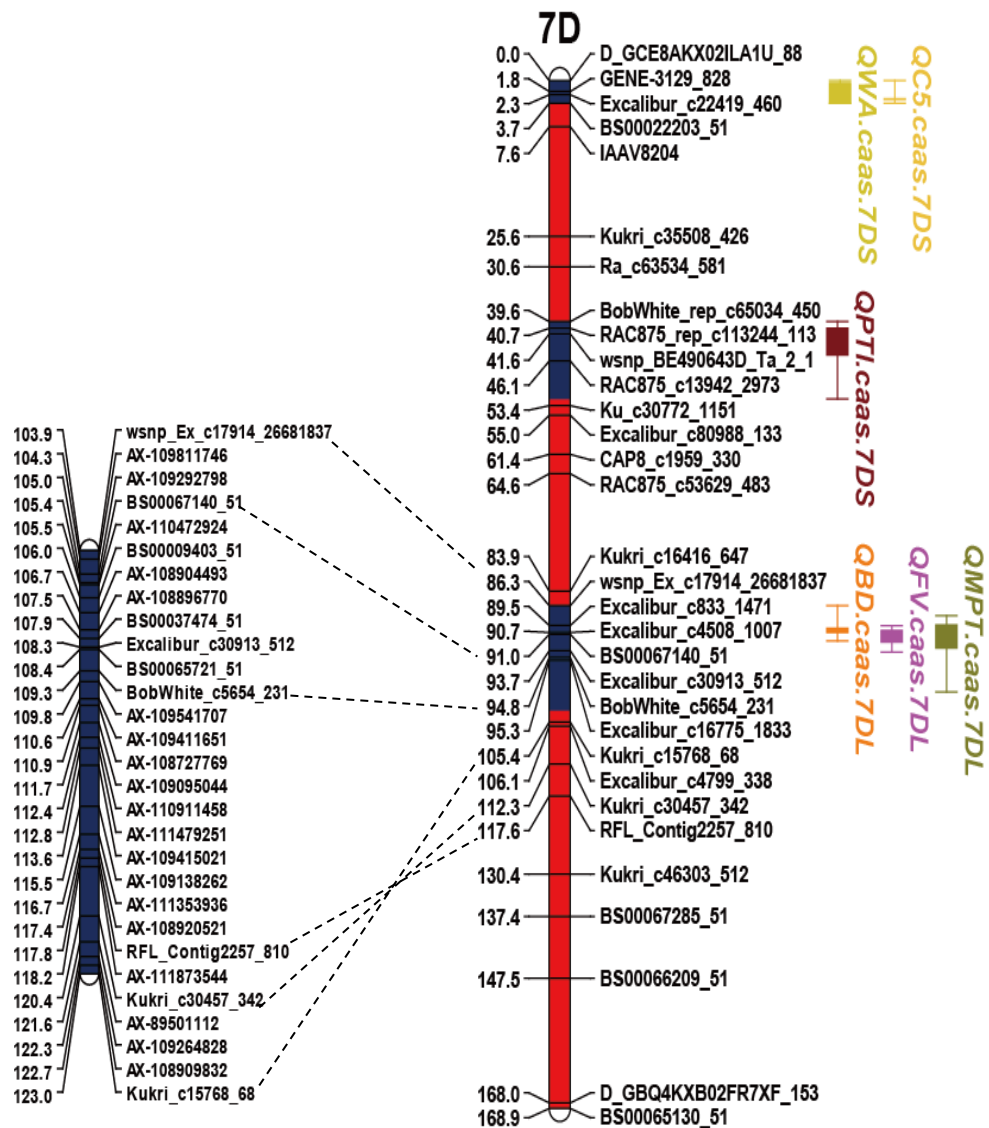

**Figure S2**

Supplement: Figure S2 — The marker-densities in regions surrounding important QTLs (QC3.caas.3AS.2 and QMPV.caas.5AL) and QTL clusters (4B, 5AS, 5BL, and 7DL) were significantly increased by the high-density linkage map. See footnote of Table 1 for abbreviations. [file Image2.PDF]

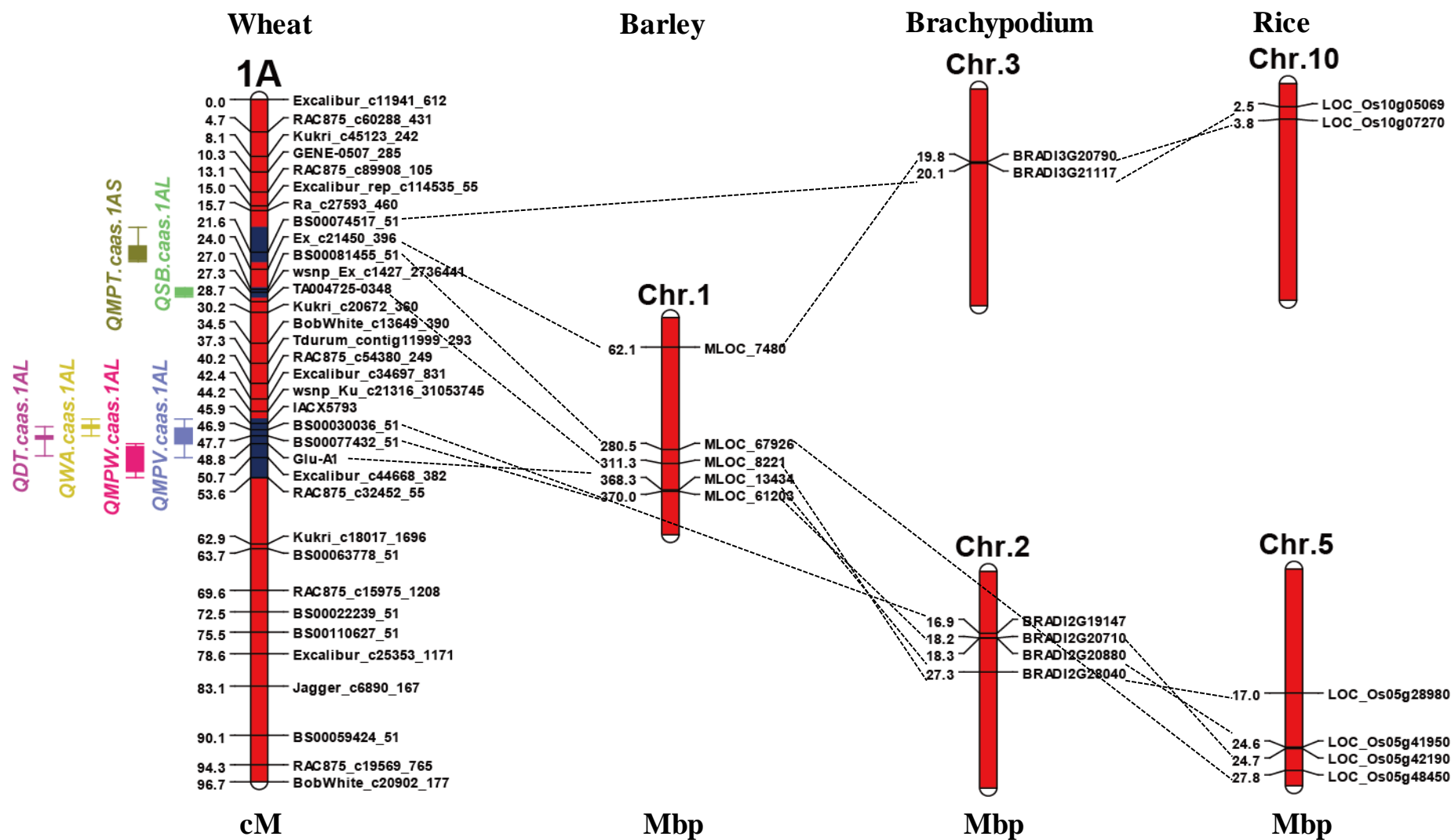

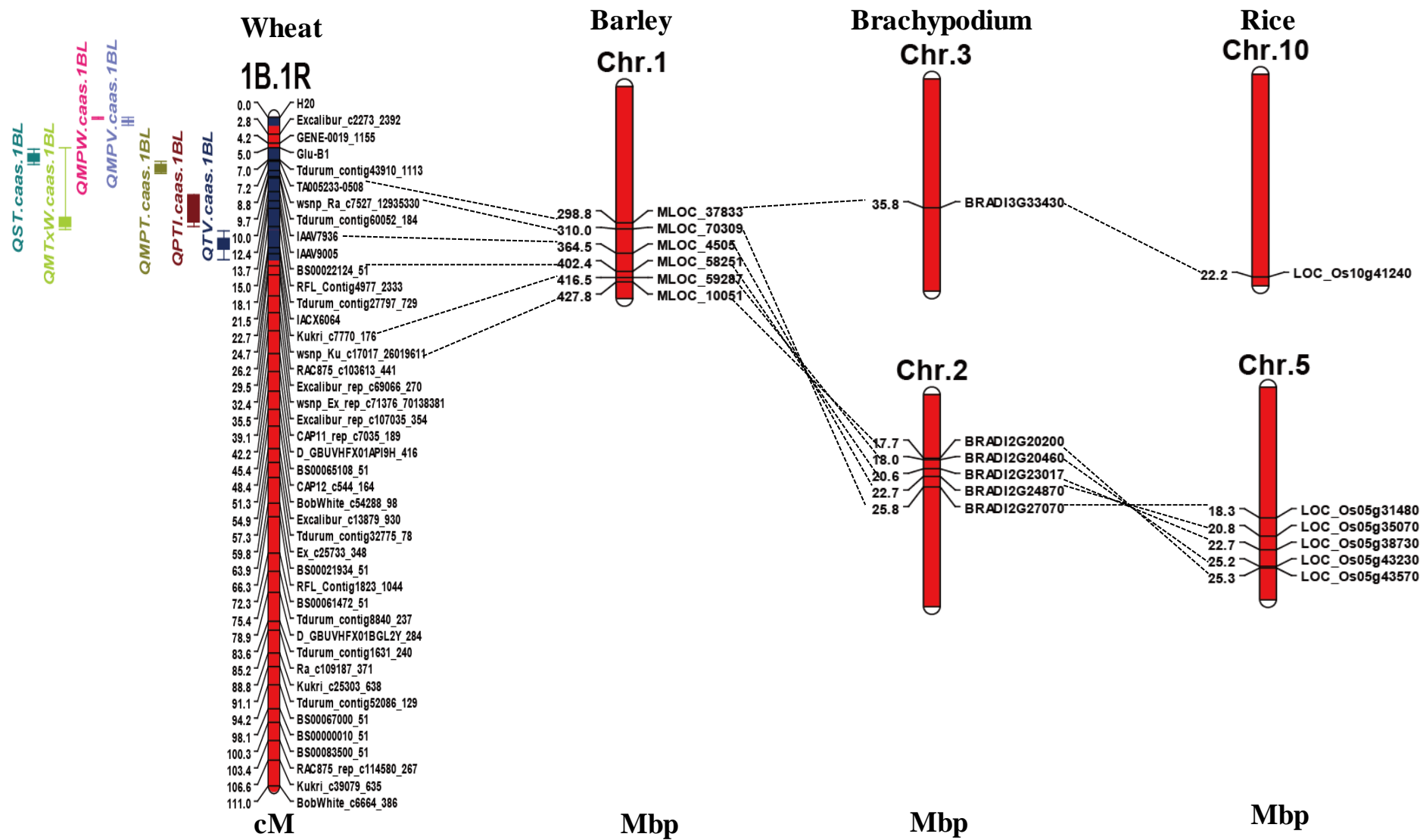

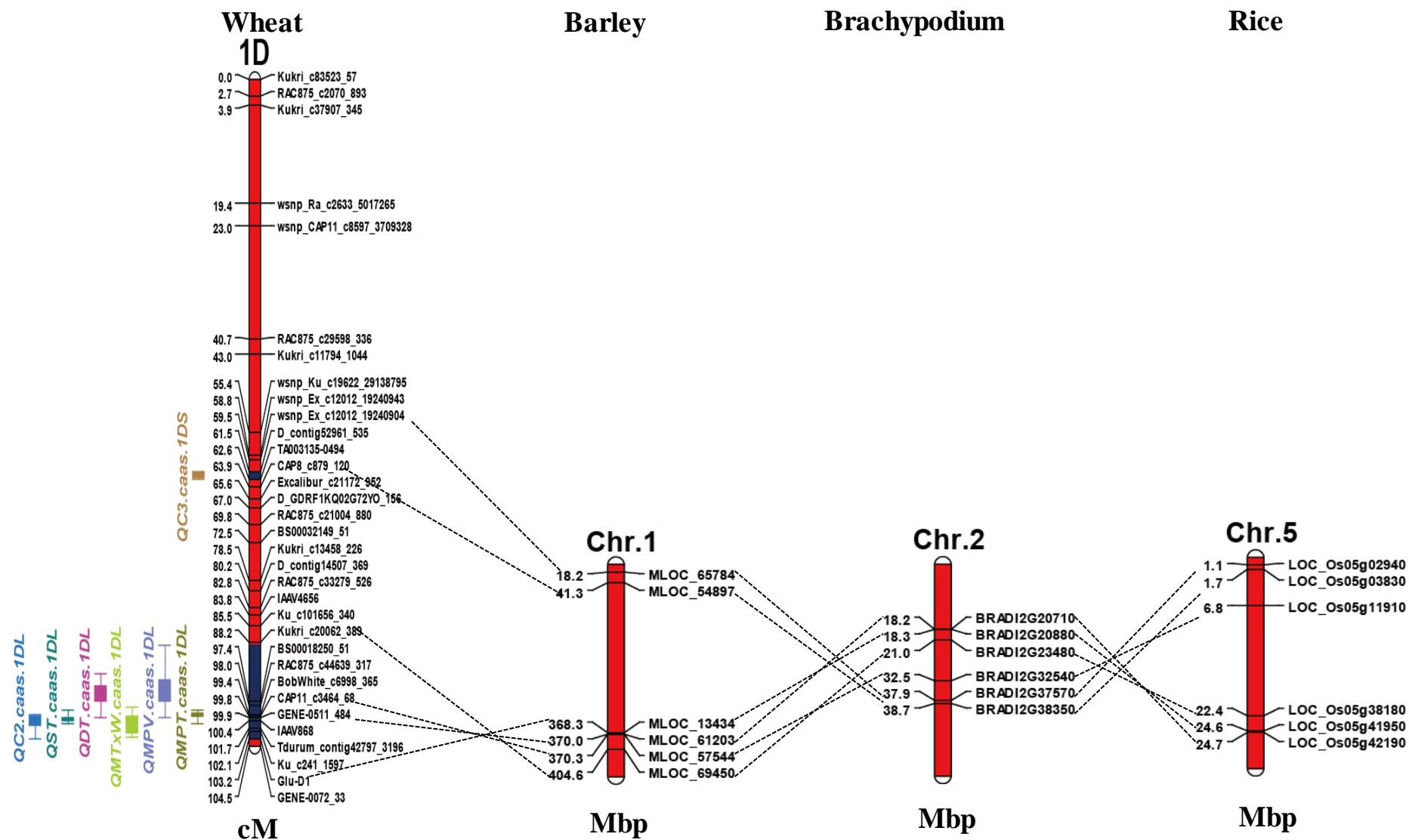

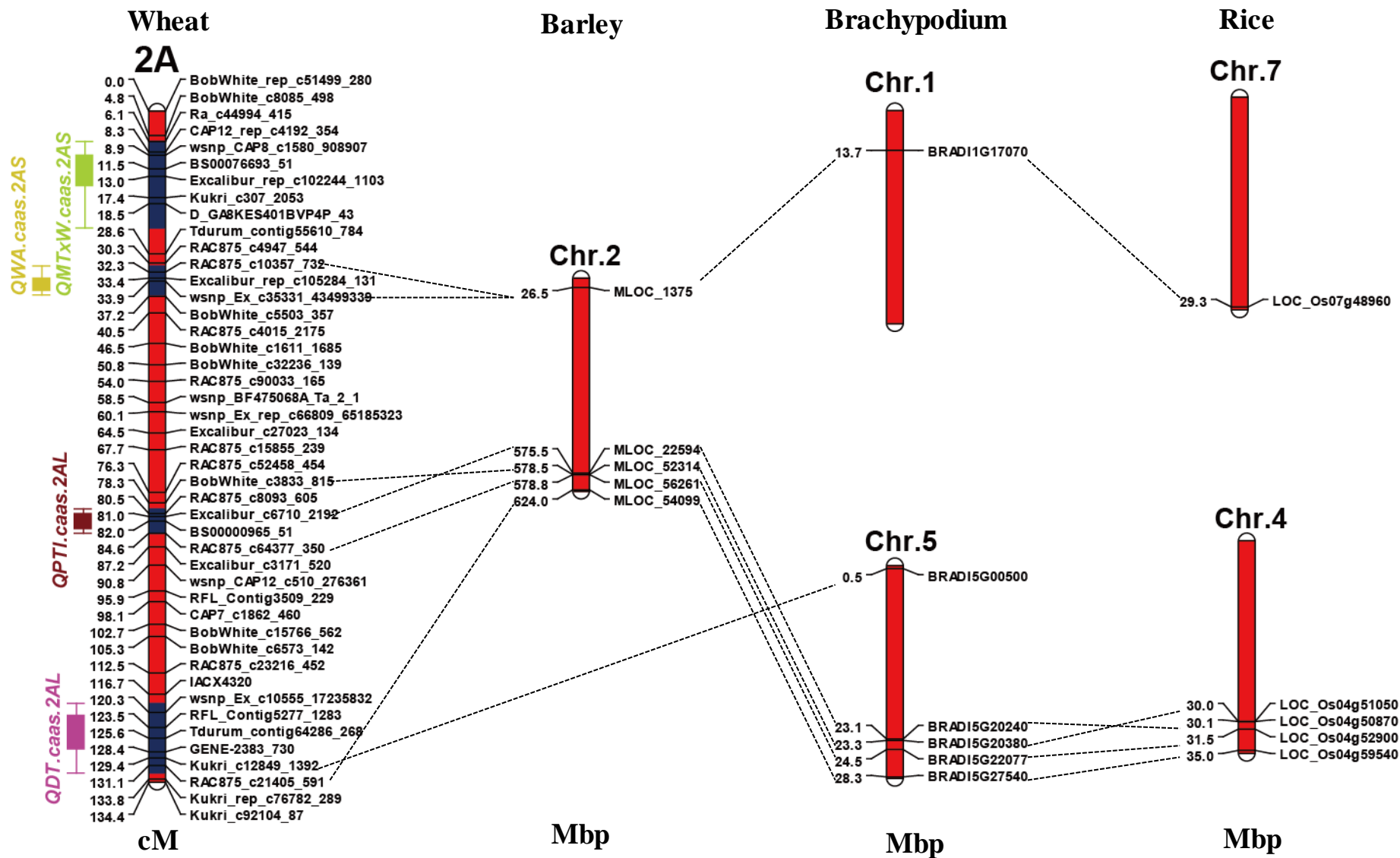

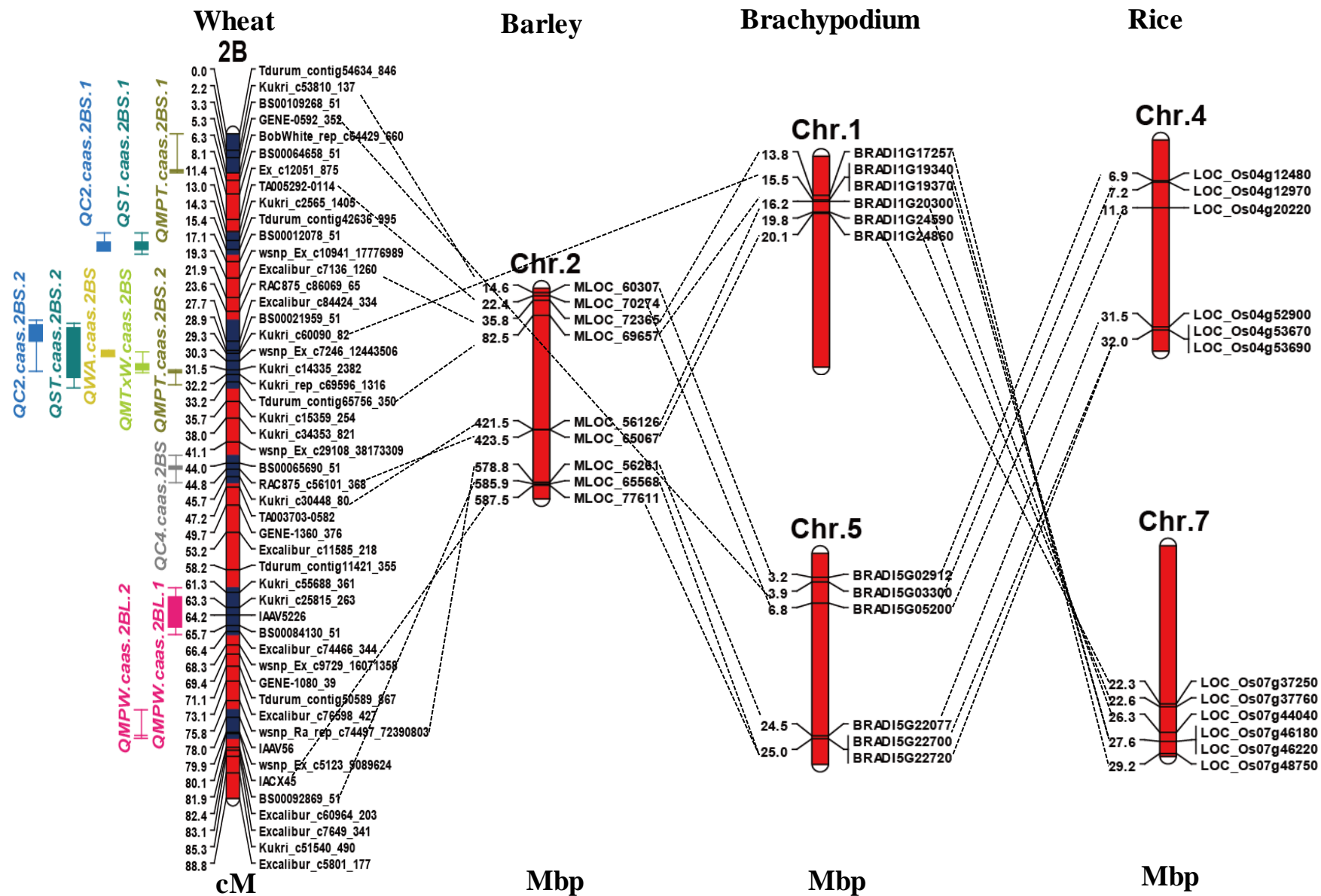

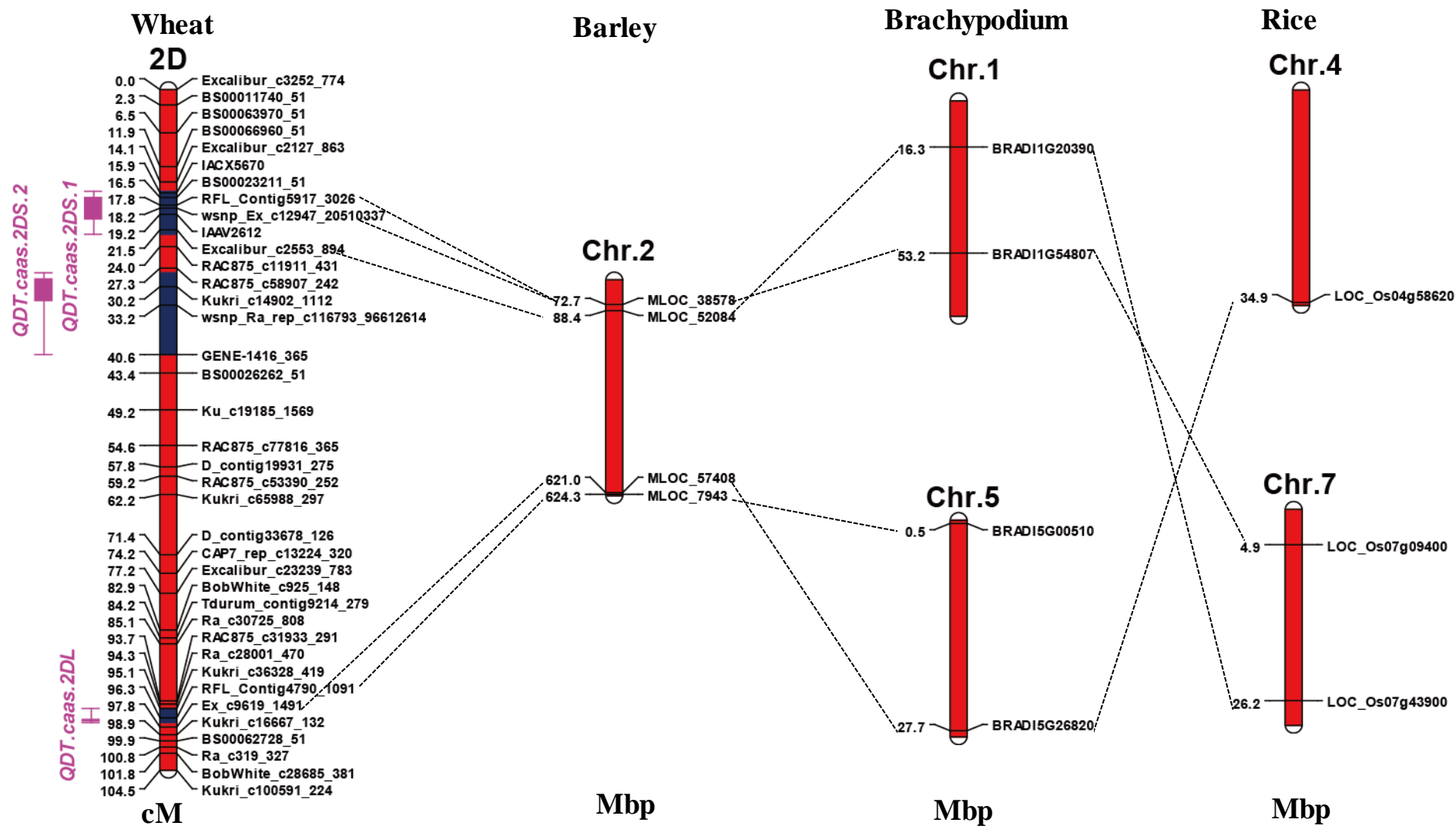

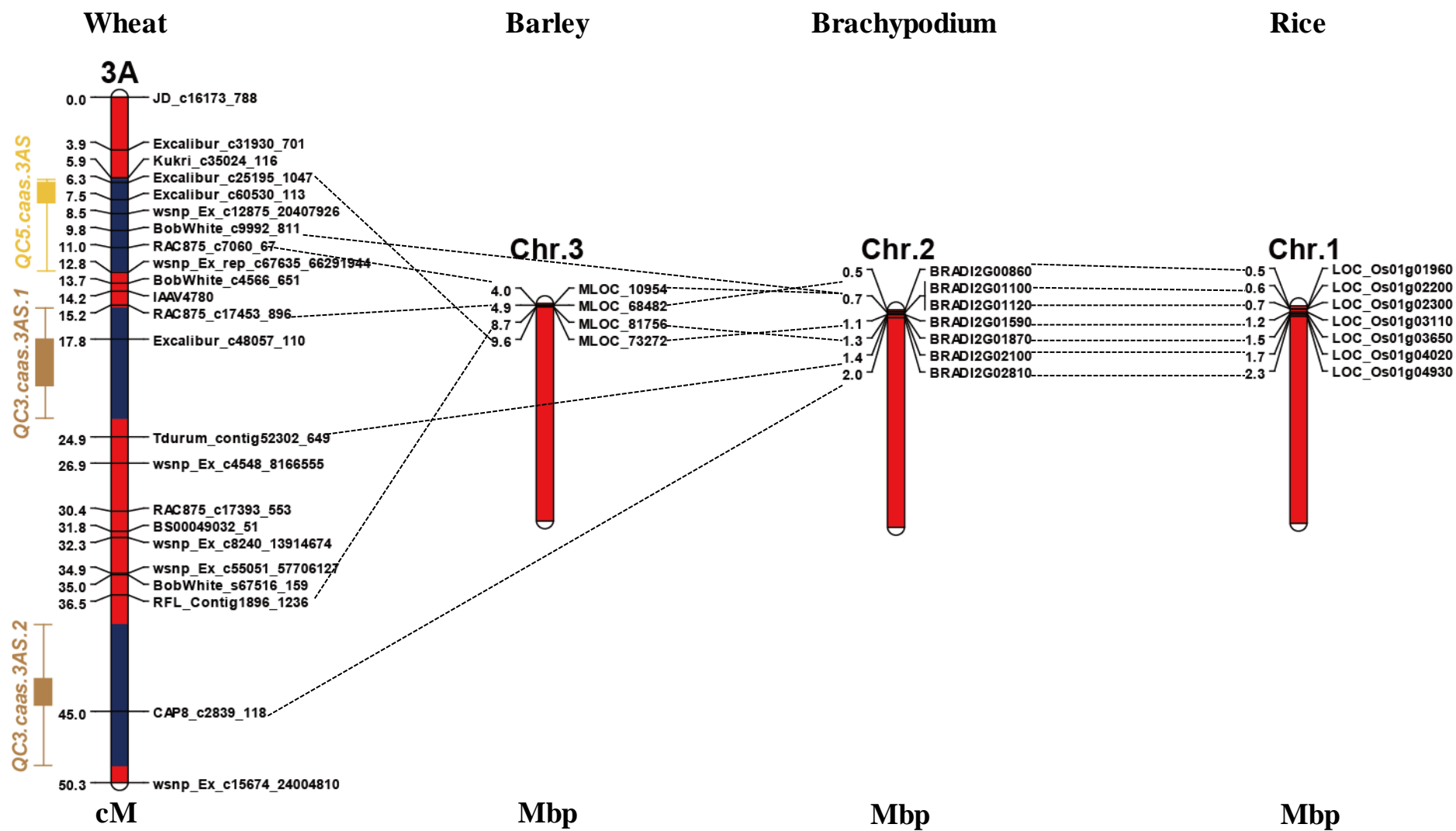

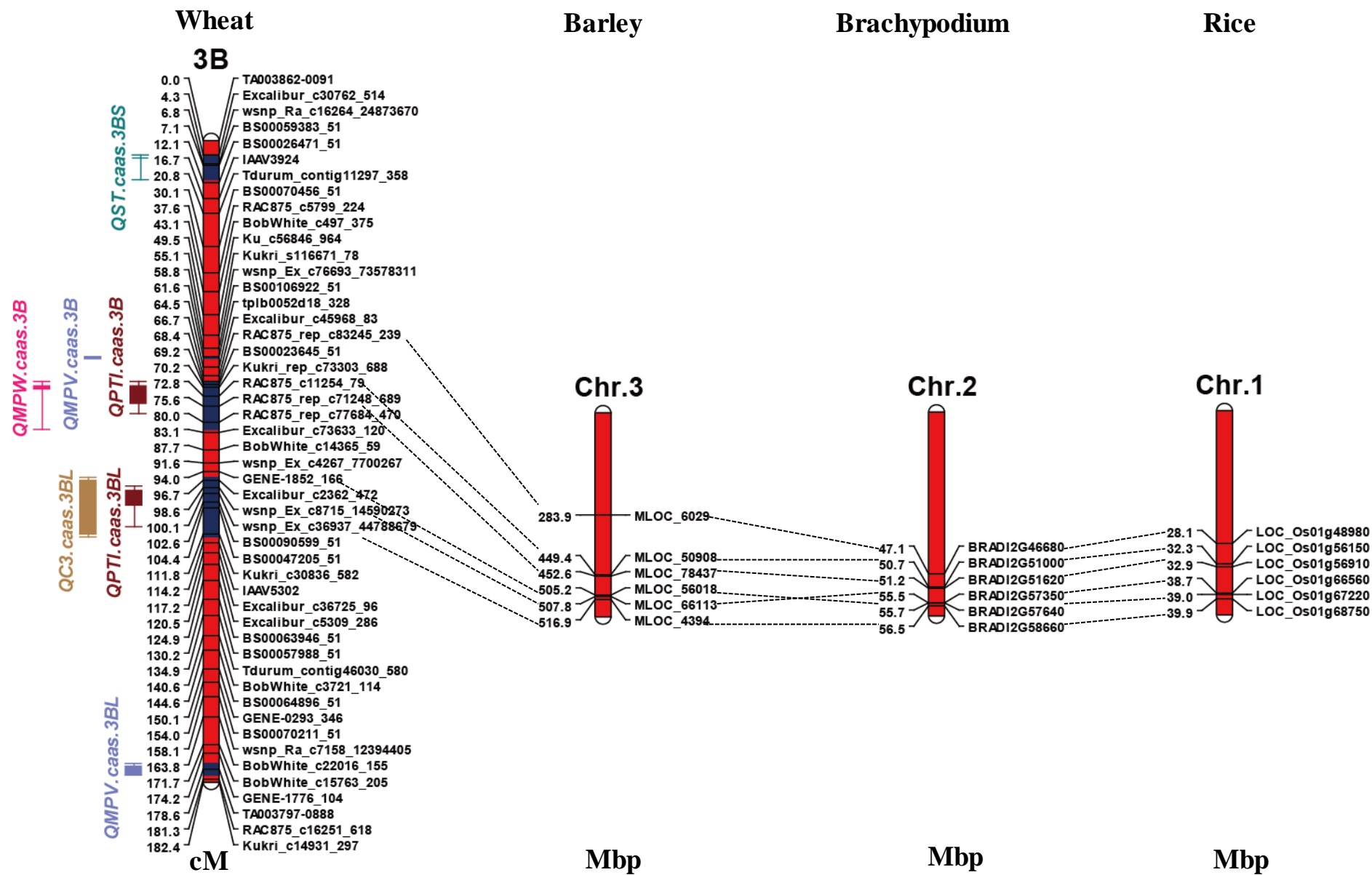

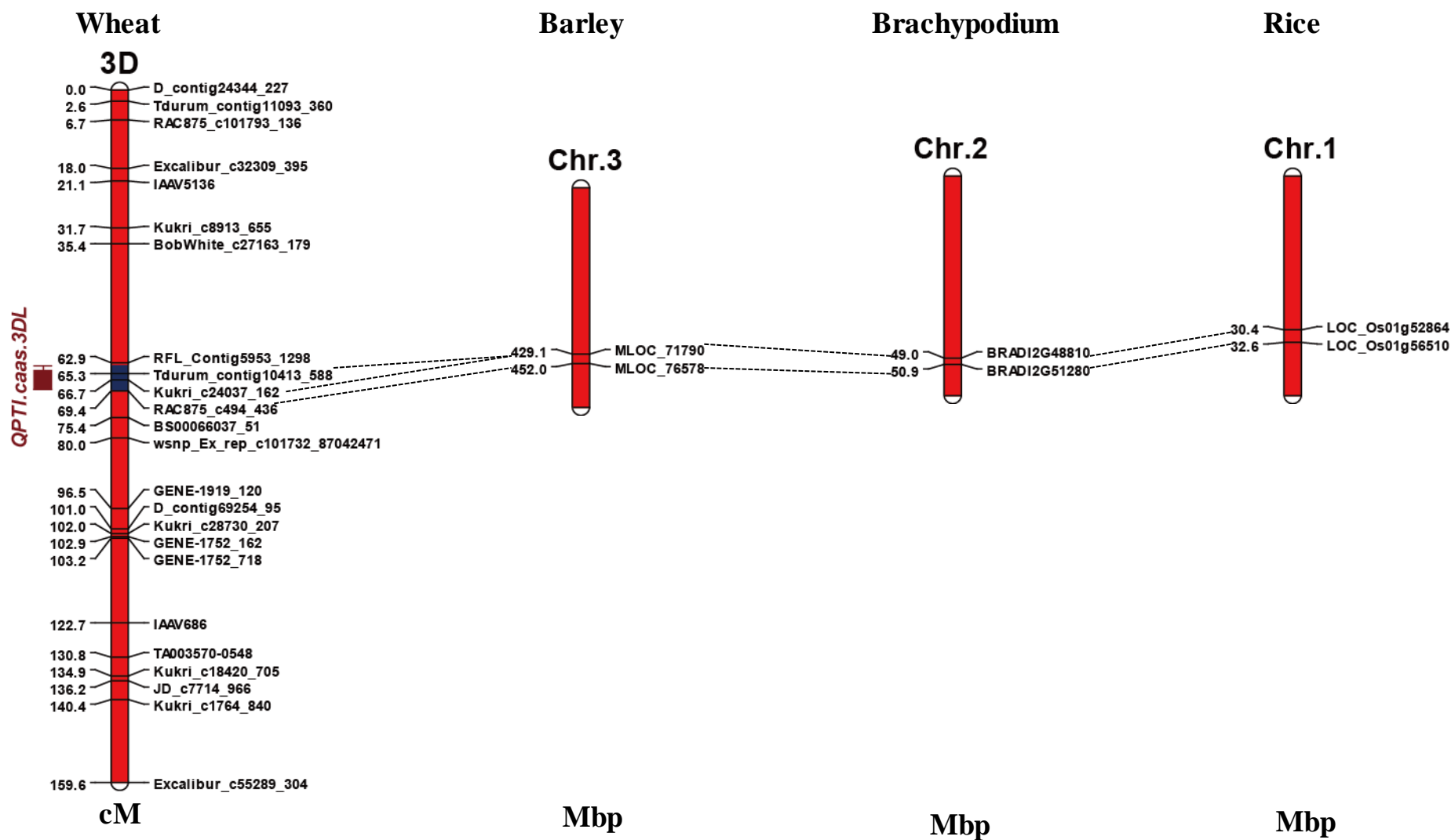

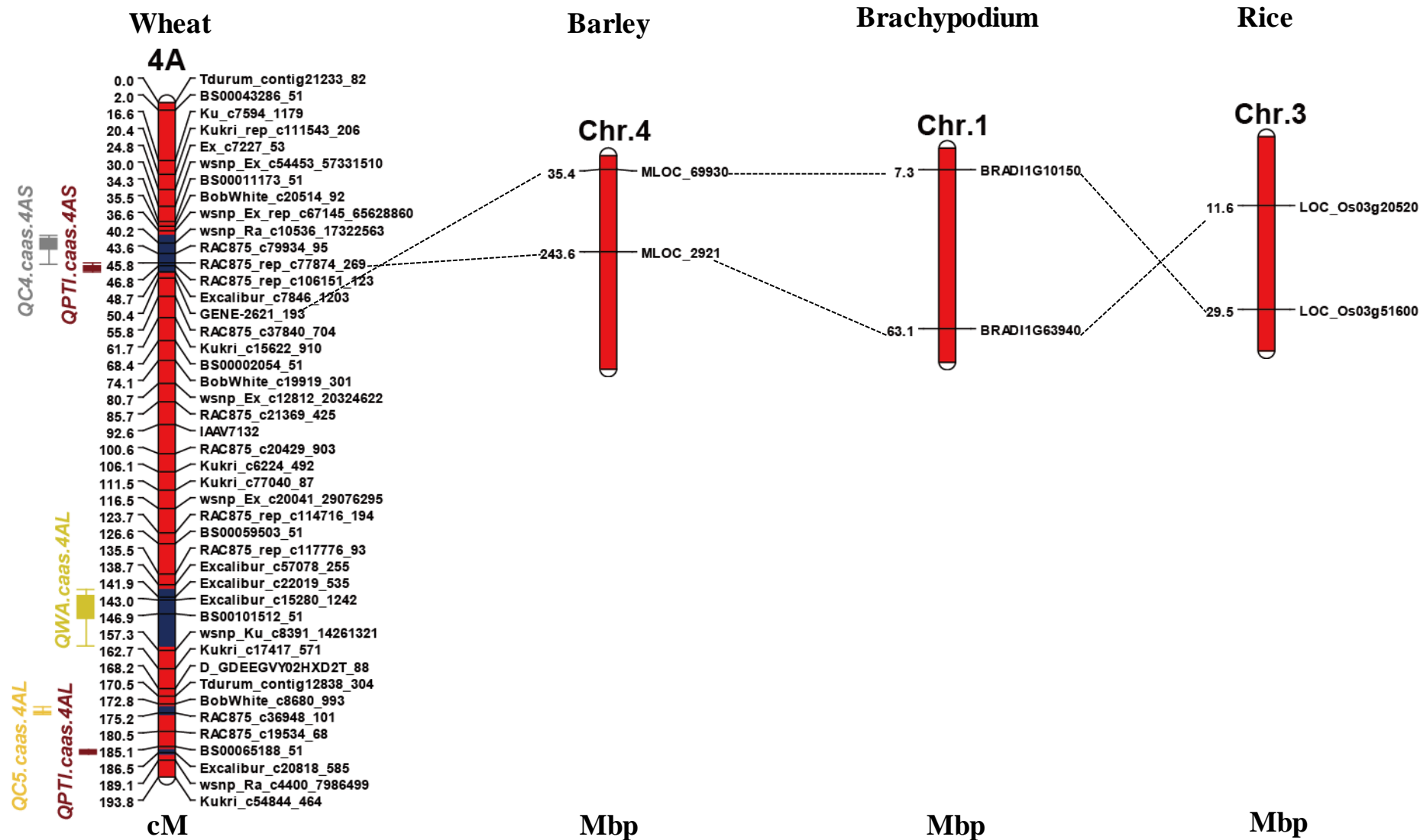

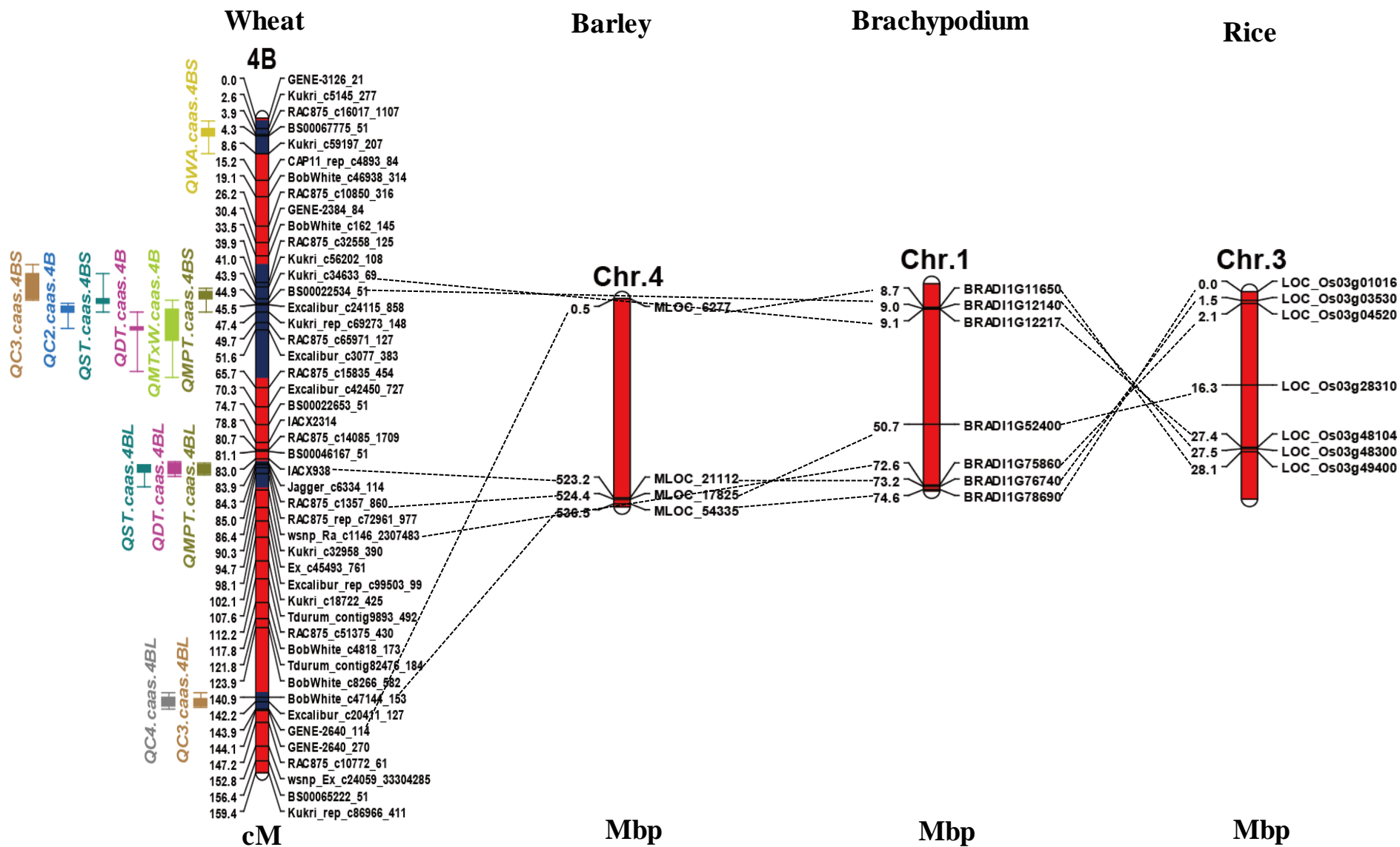

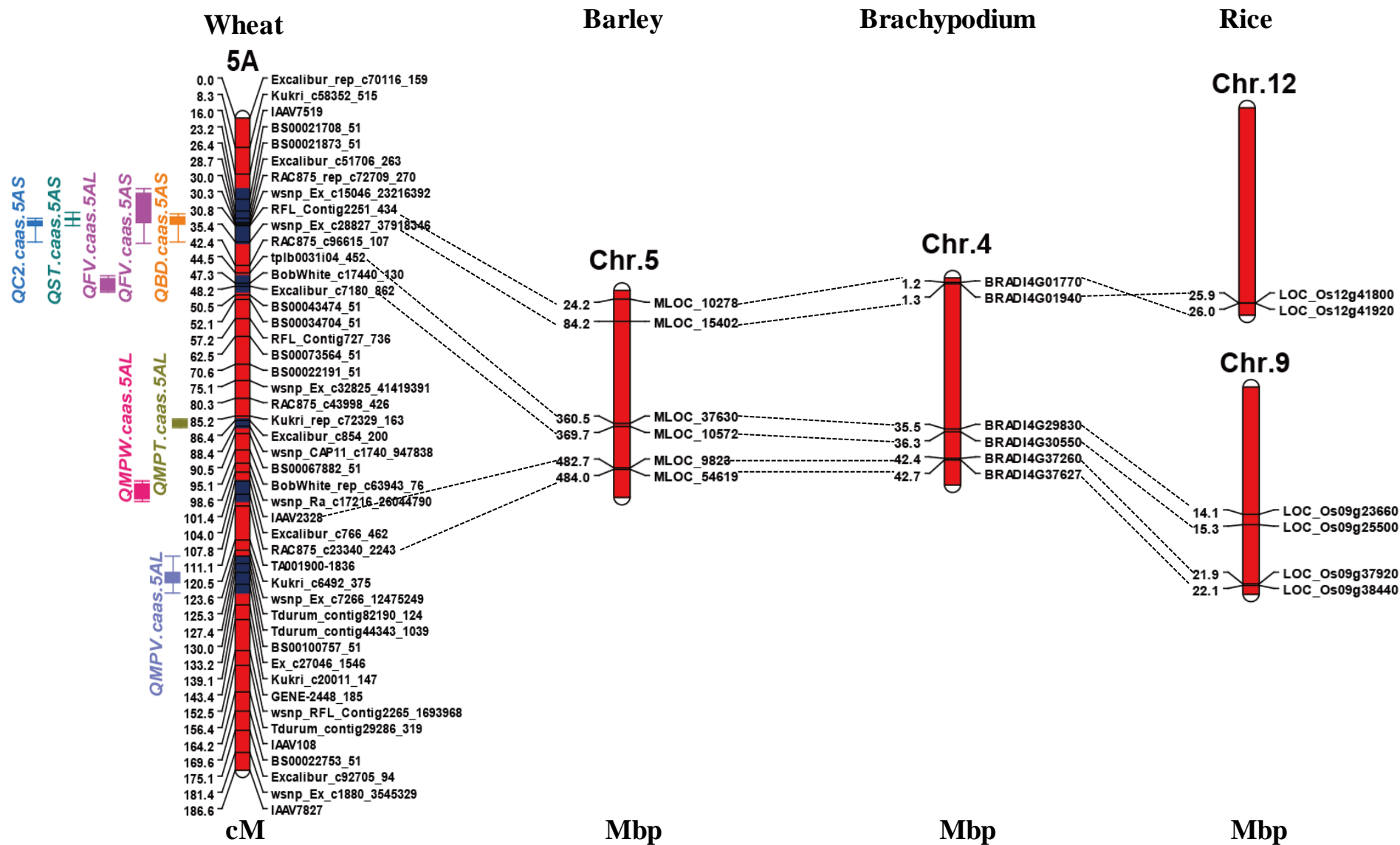

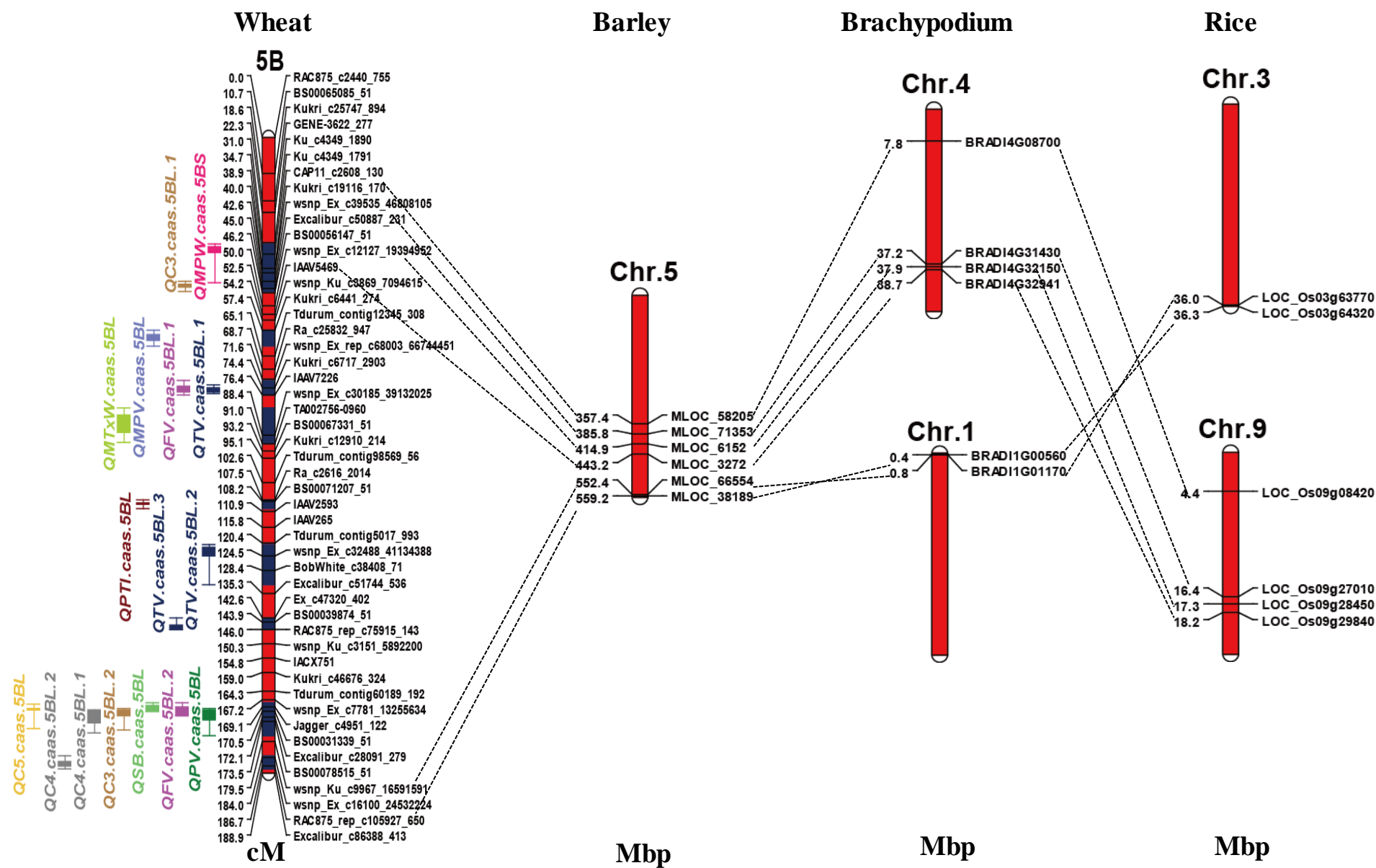

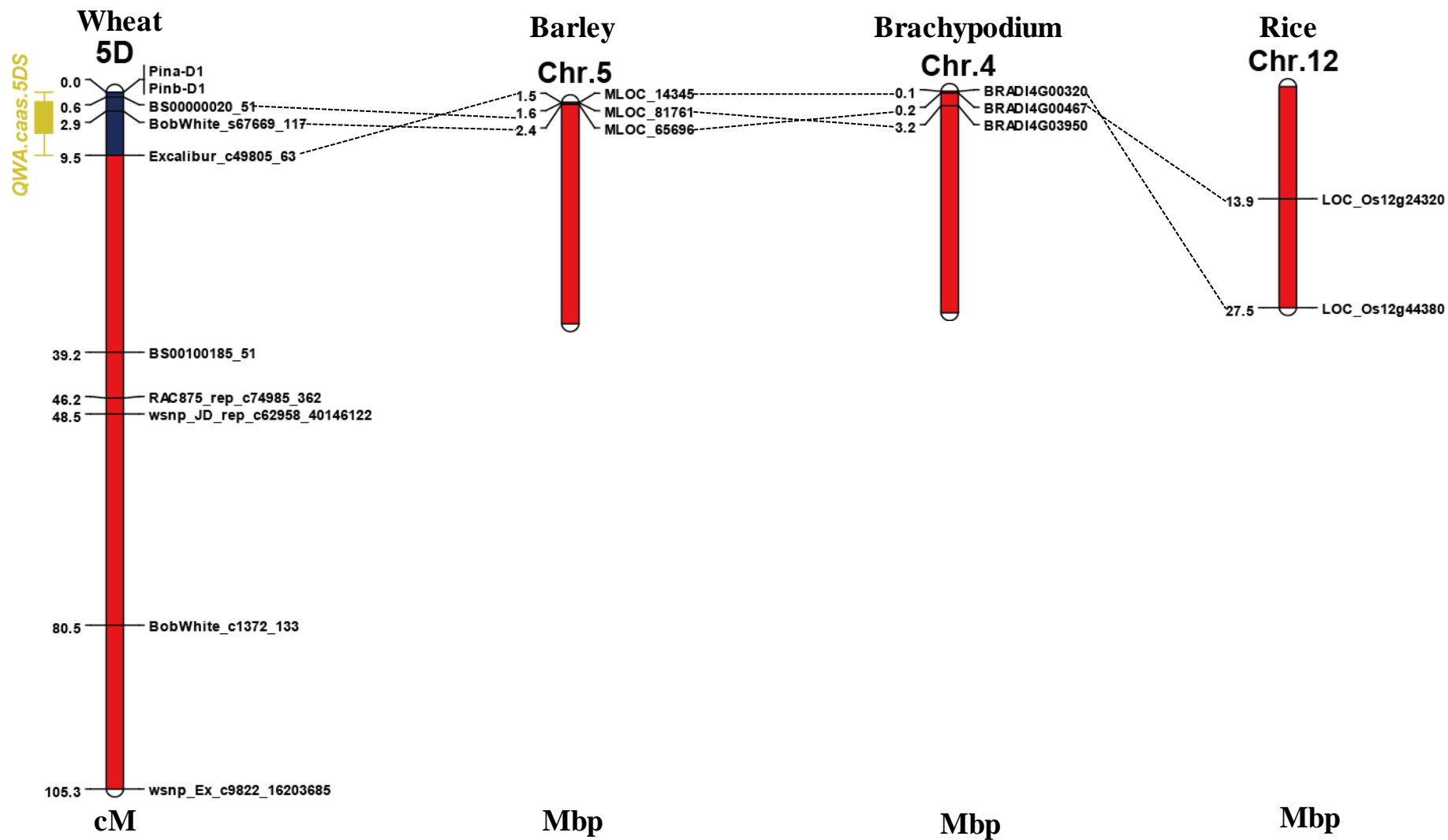

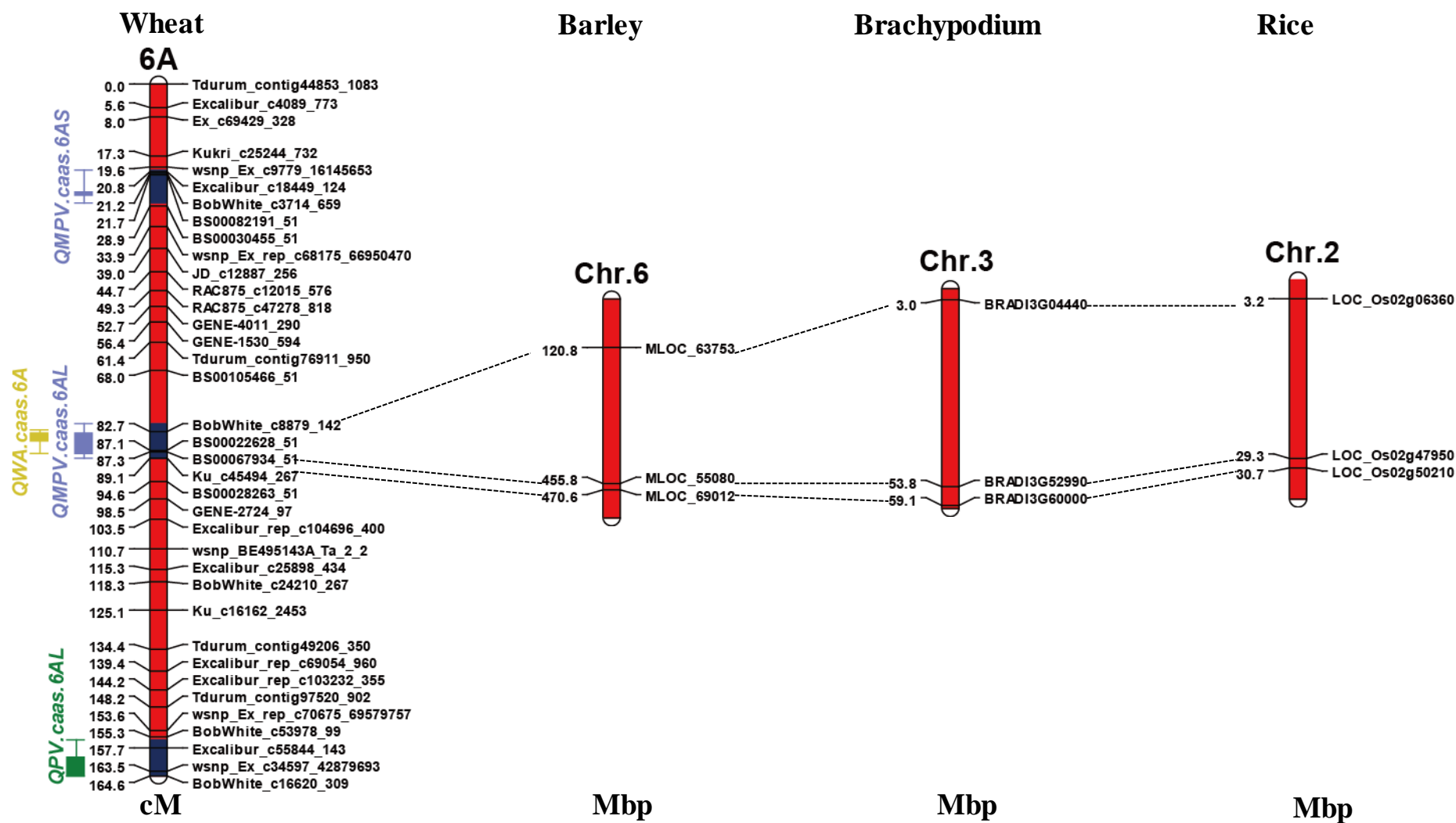

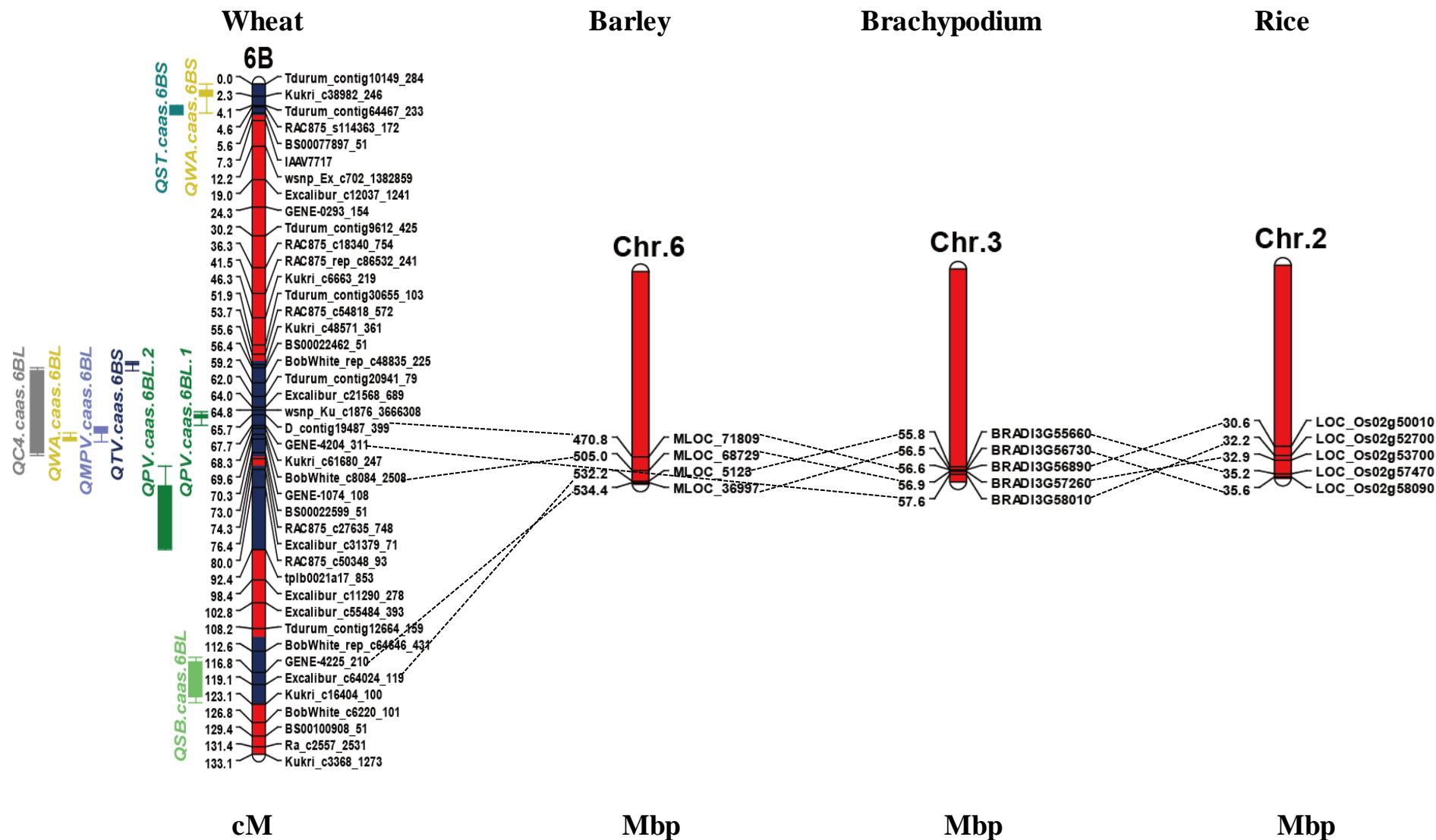

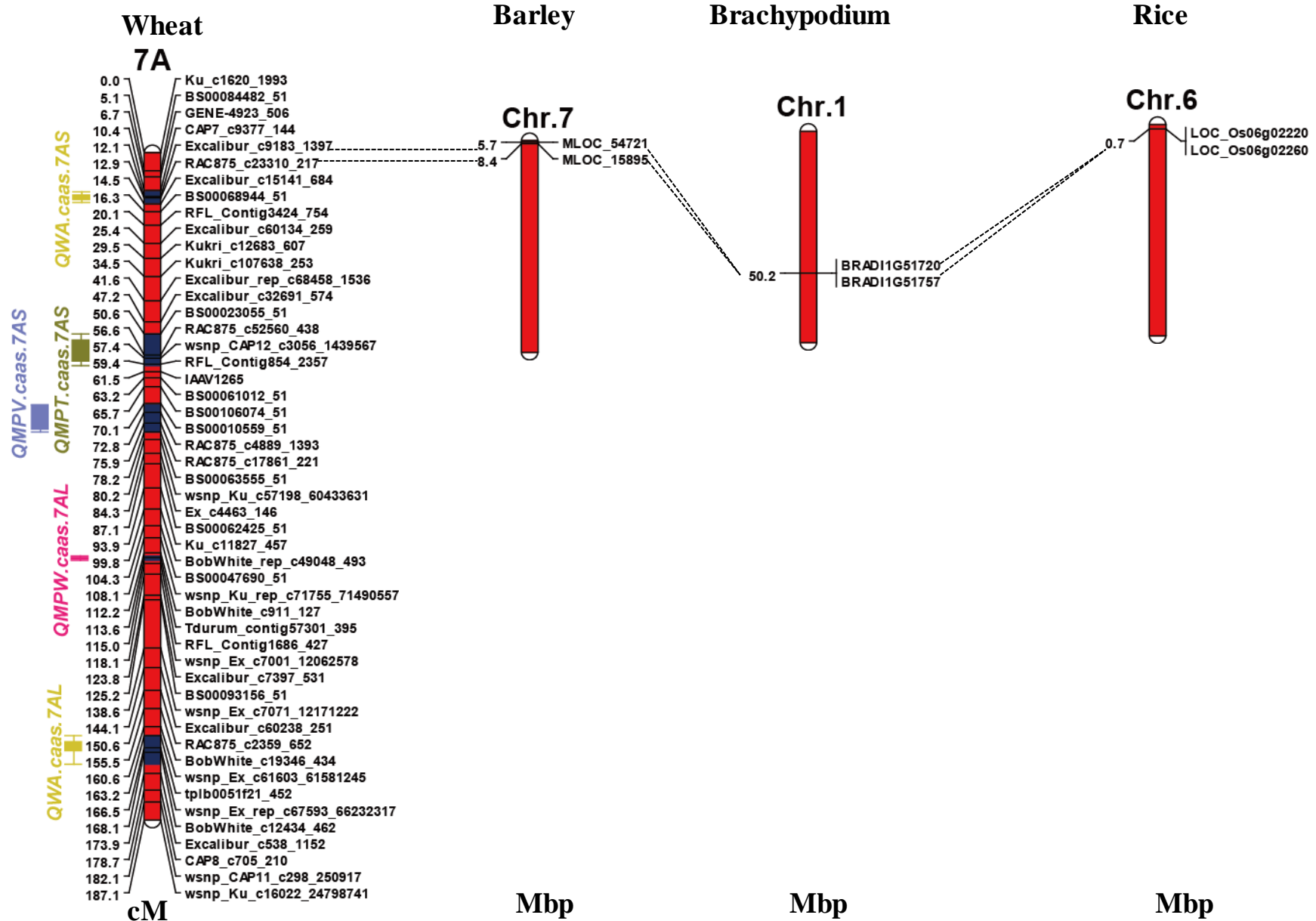

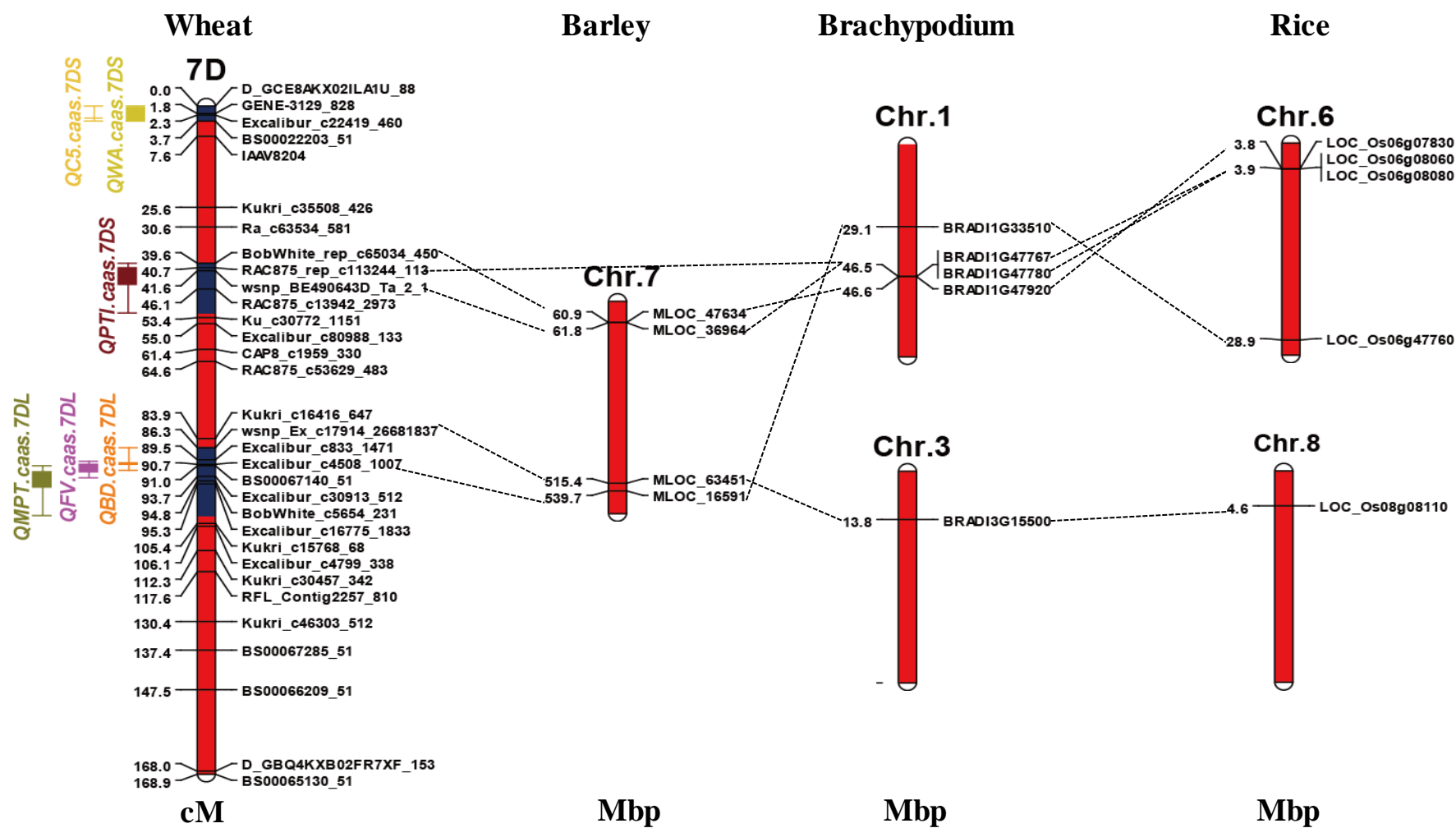

Figure S32

Supplement: Figure S3 — Comparative genomics analysis of QTLs detected in wheat (Triticum aestivum L.) with barley (Hordeum vulgare L.), brachypodium (Brachypodium distachyum L.), and rice (Oryza sativa L.). See footnote of Table 1 for abbreviations. [file Image3.PDF]
