# Supplementary material for: Genome-Wide QTL Mapping for Wheat Processing Quality Parameters in a Gaocheng 8901/Zhoumai 16 Recombinant Inbred Line Population
Source: Front Plant Sci. 2016 Jul 19;7:1032. doi: 10.3389/fpls.2016.01032 (PMC4949415; doi:10.3389/fpls.2016.01032)
Supplement: Table S1 — PCR primers for gene-specific markers used for genotyping the parents and RILs. [file Table1.DOCX]

Table S1 PCR primers for gene-specific markers used for genotyping the parents and RILs

|  | Locus | Allele/Subunit | Sequence (5’→3’) | Fragment size (bp) | Reference |
| --- | --- | --- | --- | --- | --- |
| HMW-GS | *Glu-A1* | Ax2^*^ | ATGACTAAGCGGTTGGTTCTT | 1319 | Ma et al. 2003 |
|  |  |  | ACCTTGCTCCCCTTGTCTTT |  |  |
|  |  | AxNull | ACGTTCCCCTACAGGTACTA | 920 | Lafiandra et al. 1997 |
|  |  |  | TATCACTGGCTAGCCGACAA |  |  |
|  | *Glu-B1* | By8 | TTAGCGCTAAGTGCCGTCT | 527 | Lei et al. 2006 |
|  |  |  | TTGTCCTATTTGCTGCCCTT |  |  |
|  |  | By9 | TTCTCTGCATCAGTCAGGA | 707/662 | Lei et al. 2006 |
|  |  |  | AGAGAAGCTGTGTAATGCC |  |  |
|  | *Glu-D1* | *Dx5* | GGGACAATACGAGCAGCAAA | 281 | Liu et al. 2008 |
|  |  | *Dx2* | CTTGTTCCGGTTGTTGCCA | 299 |  |
|  |  | *Dy10* | GTTGGCCGGTCGGCTGCCATG | 576 | Smith et al. 1994 |
|  |  | *Dy12* | TGGAGAAGTTGGATAGTACC | 612 |  |
| LMW-GS | *Glu-A3* | *Glu-A3a* | AAACAGAATTATTAAAGCCGG | 529 | Wang et al. 2010 |
|  |  |  | GGTTGTTGTTGTTGCAGCA |  |  |
|  |  | *Glu-A3ac* | AAACAGAATTATTAAAGCCGG | 573 | Wang et al. 2010 |
|  |  |  | GTGGCTGTTGTGAAAACGA |  |  |
|  |  | *Glu-A3g* | AAACAGAATTATTAAAGCCGG | 1345 | Wang et al. 2010 |
|  |  |  | AAACAACGGTGATCCAACTAA |  |  |
|  | *Glu-B3* | *Glu-B3d* | CACCATGAAGACCTTCCTCA | 662 | Wang et al. 2009 |
|  |  |  | GTTGTTGCAGTAGAACTGGA |  |  |
|  | 1B**_·_**1R | *Glu-B3j* | GGAGACATCATGAAACATTTG | 1500 | Francis et al. 1995 |
|  |  |  | CTGTTGTTGGGCAGAAAG |  |  |
| Waxy | *Wx-A1* | *Wx-A1a* | TCGTGTTCGTCGGCGCCGAGATGG | 389 | Nakamura et al. 2002 |
|  |  | *Wx-A1b* | CCGCGCTTGTAGCAGTGGAAGTACC | 370 |  |
|  | *Wx-B1* | *Wx-B1a* | CTGGCCTGCTACCTCAAGAGCAACT | 778 | Saito et al. 2009 |
|  |  |  | GGTTGCGGTTGGGGTCGATGAC |  |  |
|  |  | *Wx-B1b* | CGTAGTAAGGTGCAAAAAAGTGCCACG | 668 |  |
|  |  |  | ACAGCCTTATTGTACCAAGACCCATGTGTG |  |  |
|  | *Wx-D1* | *Wx-D1a* | CTGGCCTGCTACCTCAAGAGCAACT | 2307 | Nakamura et al. 2002 |
|  |  | *Wx-D1b* | CTGTTTCACCATGATCGCTCCCCTT | 1731 |  |
| Puroindoline | *Pina* | *Pina-D1b* | AATACCACATGGTTCTAGATACT | 776 | Chen et al. 2010a |
|  |  |  | GCAATACAAAGGACCTCTAGATT |  |  |
|  | *Pinb* | *Pinb-D1b* | ATGAAGGCCCTCTTCCTCA | 250 | Chen et al. 2006 |
|  |  |  | CTCATGCTCACAGCCGCT |  |  |
|  | *Pinb-2* | *Pinb-2v1* | GGTTCTCAAAACTGCCCAT | 319 | Chen et al. 2010b |
|  |  |  | ACTTGCAGTTGGAATCCAG |  |  |
|  |  | *Pinb-2v2* | CTTGTAGTGAGCACAACCTTTGCA | 401 |  |
|  |  |  | GTATGGACGAACTTGCAGCTGGAG |  |  |
|  |  | *Pinb-2v3* | GAGCACAACCTTTGCGCAATG | 398 |  |
|  |  |  | CATTAGTAGGGACGAACTTGCAGCTA |  |  |
|  |  | *Pinb-2v4* | CCTTTCTCTTGTAGTGAGCACAACCA | 403 |  |
|  |  |  | GACGAACTTGCAGTTGGAATCCAA |  |  |
