# Supplementary material for: Genome-Wide QTL Mapping for Wheat Processing Quality Parameters in a Gaocheng 8901/Zhoumai 16 Recombinant Inbred Line Population
Source: Front Plant Sci. 2016 Jul 19;7:1032. doi: 10.3389/fpls.2016.01032 (PMC4949415; doi:10.3389/fpls.2016.01032)
Supplement: Table S3 — Features of the high-density linkage map summarized by chromosome. [file Table3.DOCX]

Table S3 Features of the high-density linkage map summarized by chromosome

| Chromosome | No. of | No. of | Map length | Average distance | Average distance |
| --- | --- | --- | --- | --- | --- |
|  | markers | loci | (cM) | between markers (cM) | between loci (cM) |
| 1A | 1536 | 168 | 95.7 | 0.06 | 1.76 |
| 2A | 2238 | 239 | 258.0 | 0.12 | 0.93 |
| 3A | 871 | 136 | 127.2 | 0.15 | 1.07 |
| 4A | 6042 | 134 | 132.6 | 0.02 | 1.01 |
| 5A | 4393 | 371 | 305.9 | 0.07 | 1.21 |
| 6A | 1767 | 175 | 216.7 | 0.12 | 0.81 |
| 7A | 3165 | 380 | 303.8 | 0.10 | 1.25 |
| 1B | 2884 | 271 | 260.3 | 0.09 | 1.04 |
| 2B | 2428 | 99 | 219.8 | 0.09 | 0.45 |
| 3B | 3980 | 216 | 183.1 | 0.05 | 1.18 |
| 4B | 1422 | 206 | 192.1 | 0.14 | 1.07 |
| 5B | 6020 | 432 | 387.5 | 0.06 | 1.11 |
| 6B | 3549 | 349 | 302.5 | 0.09 | 1.15 |
| 7B | 1859 | 252 | 238.3 | 0.13 | 1.06 |
| 1D | 484 | 75 | 109.2 | 0.23 | 0.69 |
| 2D | 1865 | 217 | 235.6 | 0.13 | 0.92 |
| 3D | 399 | 67 | 78.0 | 0.20 | 0.86 |
| 4D | 429 | 91 | 117.9 | 0.27 | 0.77 |
| 5D | 43 | 21 | 91.9 | 2.14 | 0.23 |
| 6D | 930 | 52 | 78.5 | 0.08 | 0.66 |
| 7D | 657 | 107 | 186.6 | 0.28 | 0.57 |
| Group 1 | 4904 | 514 | 465.2 | 0.09 | 1.11 |
| Group 2 | 6531 | 555 | 713.3 | 0.11 | 0.78 |
| Group 3 | 5250 | 419 | 388.3 | 0.07 | 1.08 |
| Group 4 | 7893 | 431 | 442.6 | 0.06 | 0.97 |
| Group 5 | 10456 | 824 | 785.3 | 0.08 | 1.05 |
| Group 6 | 6246 | 576 | 597.7 | 0.10 | 0.96 |
| Group 7 | 5681 | 739 | 728.7 | 0.13 | 1.01 |
| Total genome A | 20012 | 1603 | 1439.8 | 0.07 | 1.11 |
| Total genome B | 22142 | 1825 | 1783.6 | 0.08 | 1.02 |
| Total genome D | 4807 | 630 | 897.7 | 0.19 | 0.70 |
| Whole Genome | 46961 | 4058 | 4121.0 | 0.09 | 0.98 |
