# Supplementary material for: Genome-Wide QTL Mapping for Wheat Processing Quality Parameters in a Gaocheng 8901/Zhoumai 16 Recombinant Inbred Line Population
Source: Front Plant Sci. 2016 Jul 19;7:1032. doi: 10.3389/fpls.2016.01032 (PMC4949415; doi:10.3389/fpls.2016.01032)
Supplement: Table S5 — SNP markers retrieved from functional genes associated with starch pasting properties. [file Table5.DOCX]

Table S5 SNP markers retrieved from functional genes associated with starch pasting properties

| Gene | Isoforms | Marker | Chromosome |
| --- | --- | --- | --- |
| ADP-glucose pyrophosphorylase (AGPase) | AGPase SS | *Ra_c29510_956* | 7AS, 7BS, 7DS |
|  |  | *Kukri_c29654_420* | 7AS, 7BS, 7DS |
|  | AGPase LS | *Kukri_rep_c97759_353* | 1AL, 1BL, 1DL |
| Granule-bound starch synthase (GBSS) | GBSS Ⅱ | *Tdurum_contig44075_1141* | 2AL, 2BL, 2DL |
|  |  | *RAC875_rep_c70705_84* | 2AL, 2BL, 2DL |
|  |  | *RAC875_rep_c69892_151* | 2AL, 2BL, 2DL |
| Soluble starch synthase (SSS) | SS Ⅰ | *JD_c2395_759* | 7AS, 7BS, 7DS |
|  |  | *JG_c4828_358* | 7AS, 7BS, 7DS |
|  |  | *BS00091891_51* | 7AS, 7BS, 7DS |
|  | SS Ⅱ | *Excalibur_rep_c109103_188* | 1AL, 1BL, 1DL |
|  |  | *Ex_c10469_1388* | 1AL, 1BL, 1DL |
|  | SS Ⅲ | *wsnp_Ex_rep_c69692_68647924* | 2AL, 2BL, 2DL |
|  |  | *wsnp_Ex_c12922_20472434* | 3B, 2AL, 2BL, 2DL |
|  |  | *wsnp_Ex_c12922_20473104* | 3B, 2AL, 2BL, 2DL |
|  | SS Ⅳ | *RAC875_c24234_199* | 1AL, 1BL, 1DL |
|  |  | *Ra_c24234_181* | 1AL, 1BL, 1DL |
| Starch-branching enzyme (SBE) | SBE Ⅰ | *wsnp_Ex_c16577_25095267* | 7AL, 7BL, 7DL |
|  |  | *IACX7421* | 7AL, 7BL, 7DL |
|  | SBE Ⅱ | *wsnp_Ku_rep_c69876_69364477* | 2AL, 2BL, 2DL |
|  |  | *wsnp_Ex_rep_c68599_67447880* | 2AL, 2BL, 2DL |
|  |  | *IAAV5656* | 2AL, 2BL, 2DL |
|  |  | *wsnp_Ex_rep_c68599_67447926* | 2AL, 2BL, 2DL |
|  |  | *wsnp_Ex_rep_c66800_65171198* | 2AL, 2BL, 2DL |
|  |  | *IAAV5669* | 2AL, 2BL, 2DL |
|  | SBE Ⅲ | *Ra_c17771_1740* | 7AL, 7BL, 7DL |
|  |  | *Ku_c11655_1866* | 7AL, 7BL, 7DL |
|  |  | *D_contig26339_350* | 7AL, 7BL, 7DL |
| Isoamylase (ISA) | ISA 1 | *RAC875_c1473_396* | 7AS, 7BS, 7DS |
|  |  | *JD_c27929_738* | 7AS, 7BS, 7DS |
|  | ISA 2 | *Kukri_rep_c101946_496* | 1AL, 1BL, 1DL |
|  |  | *JD_c4769_531* | 1AL, 1BL, 1DL |
|  | ISA 3 | *wsnp_Ex_rep_c66900_65313836* | 5AL, 5BL, 5DL |
|  |  | *wsnp_Ex_rep_c66900_65314012* | 5AL, 5BL, 5DL |
|  |  | *wsnp_Ra_c57838_59796310* | 5AL, 5BL, 5DL |
|  |  | *wsnp_Ra_c57838_59796220* | 5AL, 5BL, 5DL |
|  |  | *RAC875_rep_c113184_428* | 5AL, 5BL, 5DL |
|  |  | *Ra_c66628_801* | 5AL, 5BL, 5DL |
|  |  | *Kukri_rep_c80794_53* | 5AL, 5BL, 5DL |
| Pulllulanase (PUL) | PUL | *wsnp_Ex_c5939_10417052* | 7AS, 7BS, 7DS |
|  |  | *wsnp_Ra_rep_c69620_67130107* | 7AS, 7BS, 7DS |
|  |  | *wsnp_Ex_c24376_33619527* | 7AS, 7BS, 7DS |
|  |  | *IACX8453* | 7AS, 7BS, 7DS |
|  |  | *RAC875_c19943_2198* | 7AS, 7BS, 7DS |
|  |  | *RAC875_c19943_2021* | 7AS, 7BS, 7DS |
|  |  | *Ex_c7274_2114* | 7AS, 7BS, 7DS |
| Starh phosphorylase (Pho) | Pho 1 | *wsnp_Ra_c20970_30293227* | 5AL, 5BL, 5DL |
|  |  | *IAAV8463* | 5AL, 5BL, 5DL |
|  |  | *wsnp_Ra_c20970_30293078* | 5AL, 5BL, 5DL |
|  |  | *wsnp_Ex_c12048_19288999* | 5AL, 5BL, 5DL |
|  |  | *IAAV2219* | 5AL, 5BL, 5DL |
|  |  | *IAAV2160* | 5AL, 5BL, 5DL |
|  |  | *RAC875_c50755_244* | 5AL, 5BL, 5DL |
|  | Pho 2 | *wsnp_Ra_c21364_30746475* | 3AL, 3B, 3DL |
|  |  | *RAC875_c83597_86* | 3AL, 3B, 3DL |
|  |  | *RAC875_c44986_1396* | 3AL, 3B, 3DL |
| Disproportionating enzyme (DPE) |  | *Kukri_rep_c101462_172* | 2AS, 2BS, 2DS |
|  |  | *RAC875_c1220_153* | 2AS, 2BS, 2DS |
|  |  | *RAC875_c57419_319* | 2AS, 2BS, 2DS |
| β-Amylase (BMY) |  | *RAC875_c18908_144* | 2AS, 2BS, 2DS |
|  |  | *Kukri_s117600_90* | 2AS, 2BS, 2DS |
|  |  | *Excalibur_rep_c73324_369* | 2AS, 2BS, 2DS |
| Triose phosphate translocator (TPT) |  | *IACX5725* | 3AS, 3B, 3DS |
|  |  | *JD_c14897_71* | 3AS, 3B, 3DS |
| Sucrose transporter (SUT) |  | *BobWhite_c22549_401* | 4AS, 4BL, 4DL |
| Rice starch regulator (*TaRSR1*) |  | *RAC875_c89908_105* | 1AS, 1BS, 1DS |
|  |  | *Kukri_c40439_366* | 1AS, 1BS, 1DS |
| SPA (*OsbZIP58*) |  | *BS00050882_51* | 1AL, 1BL, 1DL |
