# Supplementary material for: Genome-Wide QTL Mapping for Wheat Processing Quality Parameters in a Gaocheng 8901/Zhoumai 16 Recombinant Inbred Line Population
Source: Front Plant Sci. 2016 Jul 19;7:1032. doi: 10.3389/fpls.2016.01032 (PMC4949415; doi:10.3389/fpls.2016.01032)
Supplement: Table S6 — Putative functions of genes corresponding to mapped SNP markers in the important QTL regions based on the linkage maps from the 90 and 660 K iSelect genotyping assays. [file Table6.DOCX]

Table S6 Putative functions of genes corresponding to mapped SNP markers in the important QTL regions based on the linkage maps from the 90K and 660K iSelect genotyping assays

| QTL | Marker^a^ | EMBL^b^ | *Tritium aestivum*^b^ | *Aegilops tauschii*^b^ | *Brachypodium*^b^ | *Triticum urartu*^b^ | Pathway |
| --- | --- | --- | --- | --- | --- | --- | --- |
| *QMPT.caas.4BL* | *Jagger_c6334_114* (1.07) | JV887782 (96.7%) |  |  | Anthranilate phosphoribosyltransferase (94%) | Anthranilate phosphoribosyltransferase, (93%) | Phenylalanine, tyrosine and tryptophan biosynthesis |
| *QMPV.caas.5AL* | *Tdurum_contig82190_124* (2.72) | JP867309 (100%) |  | Ornithine  aminotransferase (98%) | Ornithine  aminotransferase (96%) | Ornithine aminotransferase (99%) | Arginine and proline metabolism |
| *QPTI.caas.3DL* | *RAC875_c494_436* (1.71) | JW032358 (99%) |  | Lysosomal  beta glucosidase (85%) | Lysosomal  beta glucosidase (93%) |  | Starch and sucrose metabolism |
| *QWA.caas.7AS* | *CAP7_c9377_144* (1.68) | AK33416 (98%) |  | Lipoxygenase 2 (99%) |  | Lipoxygenase 2 (99%) | Linoleic acid metabolism and alpha-Linolenic acid metabolism |
| *QWA.caas.7AS* | *RAC875_c23310_217* (0.82) | JV986839 (99%) |  | 5'-methylthioadenosine/S-adenosylhomocysteine nucleosidase (99%) | 5'-methylthioadenosine/S-adenosylhomocysteine nucleosidase (91%) |  | Cysteine and methionine metabolism |
| *QC5.caas.3AS* | *wsnp_Ex_c12875_20407926* (1.24) | AF347064 (99%) | Sucrose-phosphate synthase II (98%) | Sucrose-phosphate synthase (97%) | Sucrose-phosphate synthase (90%) | Sucrose-phosphate synthase (96%) | Starch and sucrose metabolism |
| *QMTxW.caas.4B* | *AX-109563308* (1.07) | [Traes_4BS_7F31CC8A1.1](http://plants.ensembl.org/Triticum_aestivum/Transcript/Summary?db=core;g=Traes_4BS_7F31CC8A1;r=4B:8717953-8721984;t=Traes_4BS_7F31CC8A1.1;tl=3xHahA8OwJMBRSEh-4970948-86321196) (100.0%) |  |  | Glutamine amidotransferase (83%) |  | Biosynthesis of amino acids |
| *QDT.caas.4BL* | *AX-109559557* (3.00) | [Traes_4BL_27D846909.1](http://plants.ensembl.org/Triticum_aestivum/Transcript/Summary?db=core;g=Traes_4BS_7F31CC8A1;r=4B:8717953-8721984;t=Traes_4BS_7F31CC8A1.1;tl=3xHahA8OwJMBRSEh-4970948-86321196) (100.0%) |  | 3-ketoacyl-CoA synthase (83%) | 3-ketoacyl-CoA synthase (87%) |  | Fatty acid biosynthesis |

^a^Genetic distance between the peak of LOD contours and the candidate gene marker (cM)

^b^Percentage identity
